# The genetic characteristics of congenital hypothyroidism in China by comprehensive screening of 21 candidate genes

**DOI:** 10.1530/EJE-17-1017

**Published:** 2018-03-28

**Authors:** Feng Sun, Jun-Xiu Zhang, Chang-Yi Yang, Guan-Qi Gao, Wen-Bin Zhu, Bing Han, Le-Le Zhang, Yue-Yue Wan, Xiao-Ping Ye, Yu-Ru Ma, Man-Man Zhang, Liu Yang, Qian-Yue Zhang, Wei Liu, Cui-Cui Guo, Gang Chen, Shuang-Xia Zhao, Ke-Yi Song, Huai-Dong Song

**Affiliations:** 1 The Core Laboratory in Medical Center of Clinical ResearchDepartment of Endocrinology, Shanghai Ninth People’s Hospital, Shanghai Jiaotong University School of Medicine, Shanghai, China; 2 Department of EndocrinologyMaternal and Child Health Institute of Bozhou, Bozhou, China; 3 Department of EndocrinologyFujian Province Maternity & Children Hospital of Fujian Medical University, Fuzhou, Fujian Province, China; 4 Department of EndocrinologyThe Linyi People’s Hospital, Linyi, Shandong Province, China; 5 Department of EndocrinologyFujian Province Hospital, Fuzhou, Fujian Province, China; 6 Department of EndocrinologyThe People’s Hospital of Bozhou, Bozhou, Anhui Province, China

## Abstract

**Objective:**

Congenital hypothyroidism (CH), the most common neonatal metabolic disorder, is characterized by impaired neurodevelopment. Although several candidate genes have been associated with CH, comprehensive screening of causative genes has been limited.

**Design and methods:**

One hundred ten patients with primary CH were recruited in this study. All exons and exon–intron boundaries of 21 candidate genes for CH were analyzed by next-generation sequencing. And the inheritance pattern of causative genes was analyzed by the study of family pedigrees.

**Results:**

Our results showed that 57 patients (51.82%) carried biallelic mutations (containing compound heterozygous mutations and homozygous mutations) in six genes (*DUOX2*,* DUOXA2*,* DUOXA1*,* TG*, *TPO* and *TSHR*) involved in thyroid hormone synthesis. Autosomal recessive inheritance of CH caused by mutations in *DUOX2*, *DUOXA2*,* TG* and *TPO* was confirmed by analysis of 22 family pedigrees. Notably, eight mutations in four genes (*FOXE1*, *NKX2-1*, *PAX8* and* HHEX*) that lead to thyroid dysgenesis were identified in eight probands. These mutations were heterozygous in all cases and hypothyroidism was not observed in parents of these probands.

**Conclusions:**

Most cases of congenital hypothyroidism in China were caused by thyroid dyshormonogenesis rather than thyroid dysgenesis. This study identified previously reported causative genes for 57/110 Chinese patients and revealed *DUOX2* was the most frequently mutated gene in these patients. Our study expanded the mutation spectrum of CH in Chinese patients, which was significantly different from Western countries.

## Introduction

Congenital hypothyroidism (CH) is the most common neonatal metabolic disorder in the world, estimated to occur in approximately 1 in 3000–4000 live births (1). Unless treated in the first few months of life, severe CH can lead to growth retardation and permanent intellectual disability. Thus, most developed countries conduct newborn screening for CH, and all patients are diagnosed in early infancy. Newborn screening for CH have been conducted in China from 2006, the data showed that the incidence of CH was approximately 1 in 2000–2500 live births in China (2, 3, 4),which was higher compared to the worldwide levels (1/3000–1/4000). Data obtained from newborn screening have enabled us to investigate the pathogenesis of this disorder.

Most cases of CH can be classified as thyroid dysgenesis or thyroid dyshormonogenesis. Thyroid dysgenesis accounts for 80–85% of patients with primary CH and is a consequence of abnormal thyroid gland organogenesis, ranging from the lack of a thyroid gland (athyreosis) to a hypoplastic or ectopic gland (5). The following genes are associated with thyroid dysgenesis: *PAX8*, *NKX2-1*, *FOXE1*, *NKX2-5* and *HHEX* (5, 6, 7, 8). Thyroid dyshormonogenesis, which is caused by defects in thyroid hormone biosynthesis, accounts for approximately 10–15% of primary CH and is associated with the following genes: *DUOX2*,* DUOXA2*,* DUOX1*,* TPO*, *TG*,* SLC26A4*,* SLC5A5* and* TSHR* (5, 7, 9, 10, 11). A small proportion of CH patients are resistant to thyroid hormone because of mutations in *THRA* (12) or *THRB* (13, 14), which encode thyroid hormone receptors or mutations in *SLC26A2* (15, 16), which encodes the thyroid hormone transporter, thereby decreasing the sensitivity of target tissues to thyroid hormone action.

Although in most cases, CH is sporadic, there have been recent advances in elucidating its inheritance pattern. Iodine organification defects leading to goitrous hypothyroidism are inherited in an autosomal recessive manner (5, 17). Thyroid dysgenesis, which is generally thought to be sporadic, appears to have a genetic component; however, the inheritance mode is unclear and monogenic, polygenic and multifactorial inheritance have all been proposed (6, 7, 18, 19).

To date, more and more monogenic forms of thyroid dysgenesis and dyshormonogenesis have been identified. However, previous studies on CH primarily focused on only one gene or several genes. Systematic and comprehensive screening for mutation patterns of these causative genes in permanent CH is limited. In the present study, we investigated the causative genes of CH by analyzing 21 potential candidate genes reported by previous studies or based on their functions. The exons and exon–intron boundaries of these genes were analyzed by next-generation sequencing to examine mutation spectrum in 110 Chinese patients with permanent CH, and family study was conducted to investigate the inheritance pattern of causative genes.

## Subjects and methods

### Subjects

Totally, 110 Chinese patients with CH (58 females and 52 males) from non-consanguineous families, from Fujian province, Anhui province, Jiangsu province and Shanghai were recruited for this study (Table 1), including 37 family trios (proband and both parents). All patients had undergone newborn screening for CH. In brief, blood specimens from a heel prick were collected on filter paper within 3–5 days after birth, and thyroid-stimulating hormone (TSH) levels in the dried blood spots were measured by using a time-resolved fluorescence-based assay (PerkinElmer). For infants with elevated TSH levels (≥10 IU/L) on initial screening, serum levels of TSH, triiodothyronine (T3), thyroxin (T4), free T3 (FT3) and free T4 (FT4) were determined by an immunochemiluminometric assay (UniCel DxI 800, Beckman, USA). The diagnosis of permanent CH was confirmed by elevated TSH levels, T4 or FT4 levels below the reference range and restoration of normal thyroid parameters after receiving replacement therapy with l-thyroxine and after stopping treatment, a rise in TSH and a drop in fT4 were observed again. The detailed clinical information of the patients with mutation was listed in Supplementary Table 1 (see section on supplementary data given at the end of this article). Written consent was obtained from parents of the patients, and the study was approved by Ethics Committee of Shanghai Ninth People’s Hospital affiliated to Shanghai JiaoTong University School of Medicine.

**Table 1:** Clinical characteristics and thyroid function test results of 110 patients with congenital hypothyroidism. Results are expressed as mean ± s.d.

	All patients (*n* = 110)	Male patients (*n* = 52)	Female patients (*n* = 58)	Reference range
Age at diagnosis, days	27.40 ± 12.00	30.08 ± 10.01	24.76 ± 13.20	
FT3 (pg/mL)	2.85 ± 1.13	2.78 ± 0.94	2.90 ± 1.20	2.5–3.9
FT4 (ng/dL)	<0.64 ± 0.22	<0.69 ± 0.45	<0.60 ± 0.30	0.58–1.64
TSH (μIU/mL)	>82.80 ± 41.43	>77.76 ± 46.76	>87.57 ± 35.40	0.34–5.6

FT3, free triiodothyronine; FT4, free thyroxine; TSH, thyroid-stimulating hormone.

### DNA extraction and targeted sequencing of candidate genes

Peripheral blood was collected from patients and their parents. Genomic DNA was then prepared using the QuickGene DNA Whole Blood Kit L (Kurabo, Japan) according to the manufacturer’s protocol (20). Twelve previously reported possible causative genes for CH :*TPO* (GenBank reference sequence: NM_000547), *SLC5A5* (NM_000453), *TG* (NM_003235), *TSHR* (NM_000369), *DUOX2* (NM_014080), *DUOXA2* (NM_207581), *SLC26A4* (NM_000441), *FOXE1* (NM_004473), *PAX8* (NM_013952), *NKX2-1* (NM_001079668), *NKX2-5* (NM_004387), *IYD* (NM_001164694) and 9 genes based on their function: *DIO1* (NM_000792), *DIO2* (NM_000793), *THRA* (NM_001190918), *THRB* (NM_00125263), *DUOX1* (NM_017434), *DUOXA1* (NM_001276268), *GNAS* (NM_016592), *SLC16A2* (NM_006517) and *HHEX* (NM_002729) were selected in this study. Thus, a total of 21 genes were selected to be sequenced in our study. All exons and exon–intron boundaries of these genes were amplified by multiplex PCR using the 48 × 48 Access Array microfluidic platform (Fluidigm) according to the manufacturer’s protocol. Primers were designed by iPLEX Assay Design software (Sequenom). Deep sequencing of these amplicon libraries was carried out by using the HiSeq2500 or HiSeq3000 platform (Illumina, San Diego, CA, USA). To avoid base pair variants caused by multiplex PCR, target sequences were amplified and deeply sequenced in duplicate for each sample (21).

### Variants calling from the sequencing data generated by next-generation sequencing

**Figure 1:**
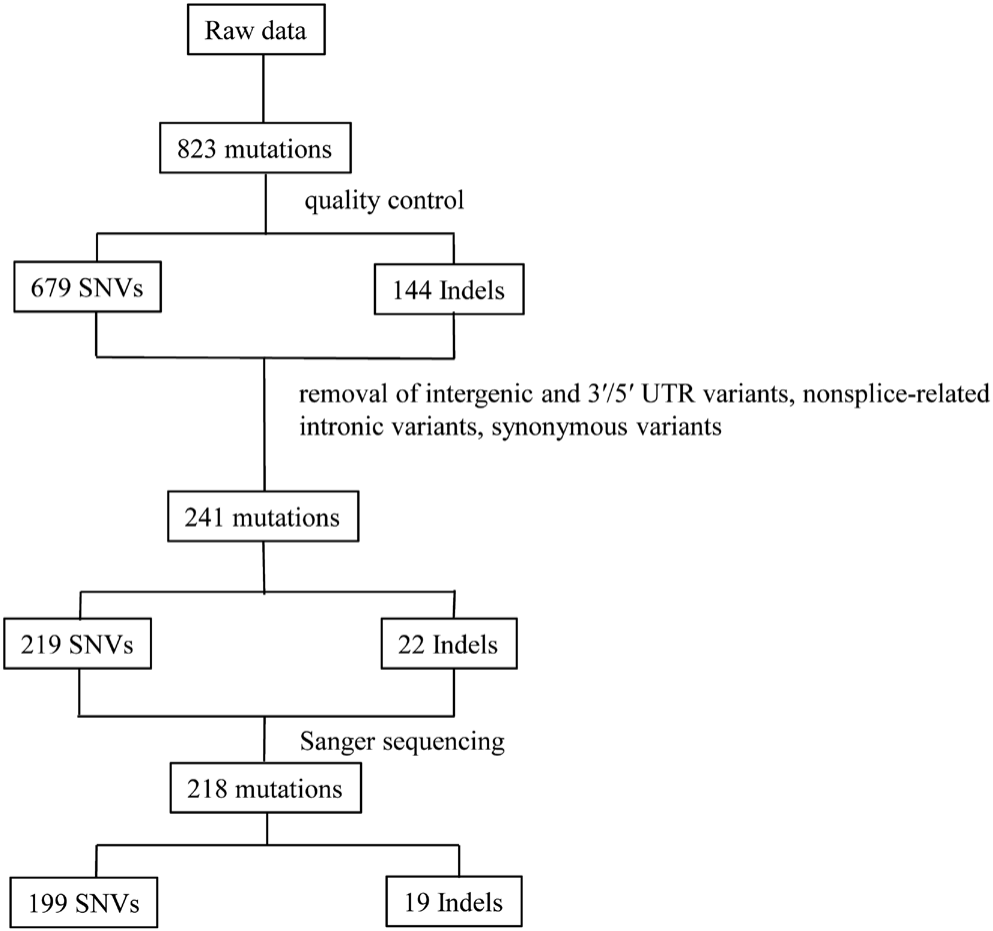
The flow chart of data analysis. The raw NGS data of 110 CH patients were generated by GATK. After quality control of raw data as described in main text, 823 relative credible mutations were selected (including 679 SNVs and 144 Indels), then intergenic and 3′/5′ UTR variants, nonsplice-related intronic variants, synonymous variants were excluded and the remaining 241 mutations (including 219 SNVs and 22 Indels) were chose to be verified by Sanger sequencing. Finally, a total of 218 mutations were verified by Sanger sequencing (including 199 SNVs and 19 Indels). SNVs, single nucleotide variants.

Raw sequence data in fastq format plus quality scores were processed as previous study (21, 22). In brief, paired-end reads were aligned to the reference human genome hg19 using the Burrows-Wheeler Alignment tool (version 0.7.7). Aligned reads were further processed by duplication removal, base quality score recalibration and indel realignment using GATK (version 2.0). Then, mappings were converted to Binary Alignment/Map file format using SAMtools (0.1.18). Initial mappings were post-processed using GATK, and the variants were considered credible if they met the following criteria: (1) quality scores for variant base pair ≥30; (2) mapping quality scores ≥50; (3) sequencing depth for variant base pair ≥20; (4) variant allele frequency ≥20%; (5) variant read depth ≥5 and (6) variant present on both strands. To exclude common single-nucleotide polymorphisms (SNP), we searched the dbSNP 135, and ESP6500 v2 databases, filtering out variants with frequencies >1% in these databases. Function was assigned using ANNOVAR based on UCSC genes. We focused on functional (protein altering) variants (removal of intergenic and 3′/5′ UTR variants, nonsplice-related intronic variants, synonymous variants) identified in duplicate samples. Then, the remaining variants were selected for validation by Sanger sequencing (Fig. 1).

## Results

### The evaluation of next-generation sequencing in 110 samples

All exons and exon–intron boundaries of 21 candidate genes were amplified by multiplex PCR using custom primers designed to generate 200–250 bp amplicons covering more than 99.5% of the target exon regions (total target bases 47 813 bp). After quality control, the average sequencing depth of these target regions was 1304× in 110 samples. All genes except three (*DIO2*, *HHEX*, *FOXE1*) had at least 80% coverage at 30× (Table 2). After bioinformatics analysis and Sanger validation, we confirmed 218 variants together (including 199 non-synonymous SNVs and 19 indels). The concordance rate between Sanger sequencing and next-generation sequencing was 90.46% (218/241). Of the non-synonymous SNVs, 90.87% (199/219) of the variants were validated, and of the indels, 86.4% (19/22) of the variants were validated (Fig. 1).

**Table 2:** Average coverage of 21 genes with sequencing depth ≥30×.

Gene	Total number of sequenced bases	Number of bases with sequencing depth ≥30×	Percentage (%)
*TPO*	2189	2135	97.52
*PAX8*	1364	1328	97.37
*DIO1*	754	732	97.07
*SLC5A5*	1947	1890	97.06
*NKX2-5*	1106	1073	97.02
*DUOX1*	4689	4535	96.71
*THRB*	1394	1347	96.63
*TG*	8355	8036	96.18
*TSHR*	2439	2342	96.01
*DUOXA1*	997	935	93.80
*GNAS*	4009	3751	93.55
*NKX2-1*	1209	1125	93.08
*THRA*	1005	933	92.86
*DUOXA2*	1432	1320	92.20
*DUOX2*	4680	4271	91.26
*SLC16A2*	1848	1686	91.22
*IYD*	1046	951	90.92
*SLC26A4*	2363	2085	88.26
*DIO2*	1045	833	79.74
*HHEX*	817	574	70.28
*FOXE1*	1123	*580*	51.69

### Mutation patterns of 21 candidate genes for CH

The 218 non-silent mutations validated by Sanger sequencing were identified in 89 of the 110 patients (80.91%, 89/110). As shown in Supplementary Table 2, the mutations were distributed in 122 sites (29 novel sites and 93 previously reported sites) of 15 genes (*DUOX1*,* DUOXA1*,* DUOX2*, *DUOXA2*,* TPO*,* SLC26A4*, *TG*,* TSHR*,* GNAS*,* HHEX*,* PAX8*, *NKX2-1*, *FOXE1, THRA* and* IYD*).

The 122 validated unique variants were classified into five categories in accordance with the American College of Medical Genetics and Genomics (ACMG) guidelines (23), namely pathogenic, likely pathogenic, variants of uncertain significance (VUS; not predicted to be damaging or the affected gene has not been previously reported with the described phenotype), likely benign or benign. Only variants classified as pathogenic or likely pathogenic are considered a ‘genetic diagnosis’ in accordance with guidelines. Of 122 validated variants, 32 variants were classified as pathogenic, 12 were likely pathogenic, 6 were likely benign and 72 variants remained VUS (Supplementary Table 2).

**Figure 2:**
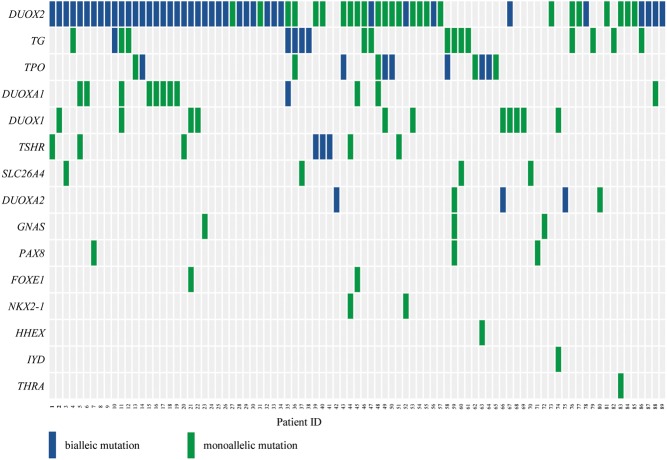
Mutations detected in 89 patients with congenital hypothyroidism. The right side were the 15 mutated genes, the bottom were the patient ID. Each column represents one patient and each row represents one gene. Blue blocks represent biallelic mutations (containing compound heterozygous mutations and homozygous mutations) and green blocks represent monoallelic mutations (heterozygous mutation). For example, patient 4 carries mutations in two genes, biallelic mutations in the *DUOX2* gene in addition to a monoallelic *TG* mutation. Patient 5 carries mutations in three genes, biallelic mutations in the *DUOX2* gene and a monoallelic mutation in *DUOXA1* in addition to a monoallelic *TSHR* mutation. A total of 57 patients carried biallelic mutations in *DUOX2*, *TG*, *TPO*, *TSHR*, *DUOXA2* or *DUOXA1*. A total of 66 patients harbored mutations in *DUOX2*, which was the most frequently mutated gene.

The most frequently mutated gene in our study was *DUOX2* (60%, 66/110), followed by *TG* (16.36%, 18/110). Notably, 57 (51.82%) of the patients carried biallelic mutations in at least of one the following genes involved in thyroid hormone synthesis: *DUOX2*,* TG*,* TPO*,* DUOXA1, TSHR* and *DUOXA2* (Fig. 2).

Eight mutations distributed in 5 mutation sites were identified in 4 transcription factor genes (*HHEX*, *FOXE1*, *NKX2-1* and *PAX8*) from eight patients (Fig. 2). These genes play key roles in thyroid development and are considered causative genes for thyroid dysgenesis. These mutations were monoallelic, which is consistent with a study by Opitz *et al*. (24). Of the two recurrent mutations in these genes, one located in *PAX8* was present in three patients, and the other located in *NKX2-1* was present in two patients. Six family trios with *FOXE1*, *NKX2-1* and *PAX8* mutations were available for analysis. The parents of probands who carried heterozygous mutations of these genes did not have hypothyroidism (Supplementary Fig. 1).

### Common mutations in Chinese patients with CH and its inheritance patterns

**Figure 3:**
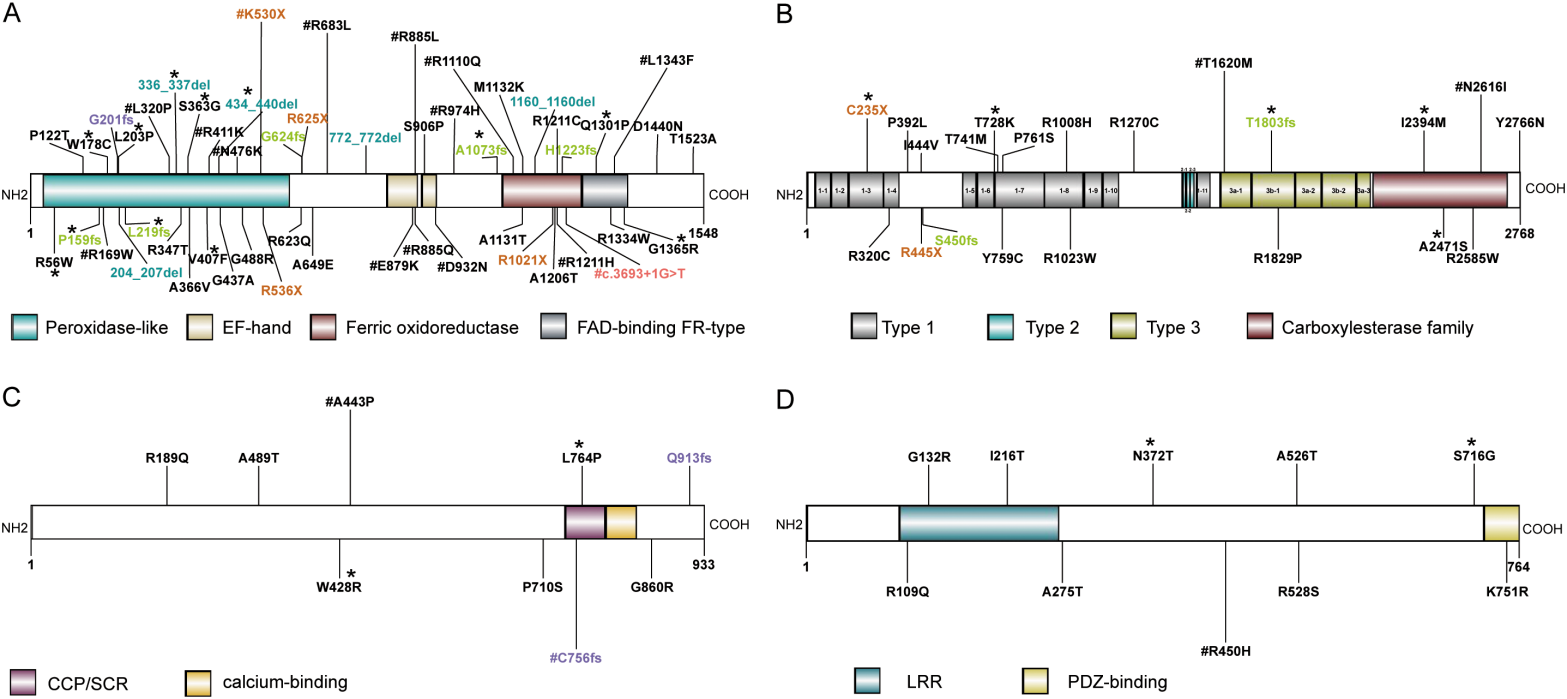
Mutation sites in the secondary structure of *DUOX2*, *TG*, *TSHR* and *TPO* proteins. (A) Fifty-one mutation sites distributed in the secondary structure of *DUOX2* protein, which contains 4 domains, peroxidase-like domain, EF-hand domain, ferric oxidoreductase domain and FAD-binding FR-type domain. A total of 41 mutations were located in these domains region. (B) Twenty-one mutation sites located in the secondary structure of* TG* protein, which include 3 domains, type 1 domain, type 2 domain, type 3 domain and carboxylesterase family domain. Fifteen mutation sites were in domain region. (C) Nine mutation sites were in *TPO* protein with CCP/SCR domain and calcium domain, only two mutations (L764P, C756fs) were in domain region. (D) Ten mutation sites were located in *TSHR* protein, of which 3 were in LRR domain and one was in PDZ-binding domain. FAD, flavin adenine dinucleotide; CCP, the complement control protein; SCR, short consensus repeat; LRR, leucine-rich repeats. Missense mutations, stopgain mutations, splicing mutations, frameshift deletion mutations, frameshift insertion mutations and nonframeshift deletion mutations are indicated with black, brown, red, green, purple and blue font, respectively. *Denotes the novel mutations and ^#^denotes the recurrent mutations.

Of the 21 candidate genes for CH analyzed in this study, the gene with the highest frequency of biallelic mutations was *DUOX2*, followed by *TPO*, *TG* and *TSHR*. We identified 120 non-silent variants of *DUOX2* distributed in 51 mutation sites in 66 patients, of which, 41 (41/110, 37.27%) carried biallelic mutations (Fig. 2). Of the 51 mutation sites of *DUOX2*, 22 were located in the peroxidase-like domain, 10 in the ferric oxidoreductase domain and 4 in the FAD-binding FR-type domain, all of which play key roles in the function of *DUOX2*. Seventeen of the 51 mutation sites were recurrent sites, and except for R974H and R683L, they were all located in functional domains. The following 12 novel mutations were identified among the 51 non-silent *DUOX2* variants: Q1301P, S363G, p.336_337del, L203P, P159fs, L219fs, A1073fs, W178C, V407F, p.434_440del, G1365R and R56W (Fig. 3A).

Thyroglobulin (*TG*) contains four similar types of cysteine-rich repeat modules and plays a key role in thyroid hormone synthesis. We identified 21 mutation sites of *TG* in 18 patients, of which, 5 (5/110, 4.5%) carried biallelic mutations (Fig. 2). The N-terminal region of human *TG* contains 11 type-1 repeats, each of which usually contains six conserved cysteines and consists of about 70 amino acids. Of the 21 non-silent *TG* variants, five were novel mutations (C235X, T728K, T1803fs, I2394M and A2471) and two were recurrent mutations (T1620M, observed in 3 patients, and N2616I, observed in 2 patients). Most non-silent variants, including the recurrent T1620M mutation, were located in cysteine-rich domains of *TG* (Fig. 3B).

Thyroid peroxidase (*TPO*) catalyzes the oxidation of iodide, iodination of specific tyrosine residues of TG and the coupling of two DIT molecules to form T4 or one DIT and one MIT to form T3. Nine non-silent variant sites in *TPO* were detected in 12 patients, of which seven patients carrying biallelic *TPO* mutations, namely two carried compound heterozygous mutations and five carried homozygous mutations (A489T, *n* = 1; A443P, *n* = 2; L764P, *n* = 1; and C583fs, *n* = 1) (Fig. 2). The recurrent mutations C756fs and A443P were identified in four patients and three patients, respectively. Of the nine mutation sites in *TPO*, only L764P and C756fs were located in CCP/SCR (CCP, the complement control protein/ SCR, short consensus repeat) domain and the novel mutations were W428R, L764P (Fig. 3C).

All steps in the formation and release of thyroid hormones are regulated by thyroid-stimulating hormone (TSH), which is produced by pituitary thyrotrophs. The TSH receptor (*TSHR*), which is expressed in thyroid cells, contains a large extracellular N-terminal domain, seven membrane-spanning domains and an intracellular domain. Ten non-synonymous SNVs were in *TSHR*, including the recurrent mutation R450H and 2 novel mutations (S716G and N372T) were identified in eight patients, of which three carried biallelic mutations (Fig. 2). Three mutation sites, namely, R109R, G132R and I216T, were located in leucine-rich repeats (LRR) domain and K751R was situated in PDZ-binding domain (Fig. 3D).

Although the mutation frequency of *DUOXA1* was high (12/110), only one patient (patient 35) carried a biallelic mutation in *DUOXA1*. In contrast, three of the five patients with *DUOXA2* mutations carried the biallelic mutations (Fig. 2). The difference of mutation frequency of *DUOXA1* and *DUOXA2*, which were the maturation factors of *DUOX1* and* DUOX2,* respectively, in 110 patients suggested *DUOX2* played a more important role in CH than *DUOX1* (11). Biallelic mutations in *TG*,* TPO*,* TSHR*,* DOUXA2* and* DOUXA1*, which are involved in thyroid hormone synthesis, were observed in 18 patients (Fig. 2).

**Figure 4:**
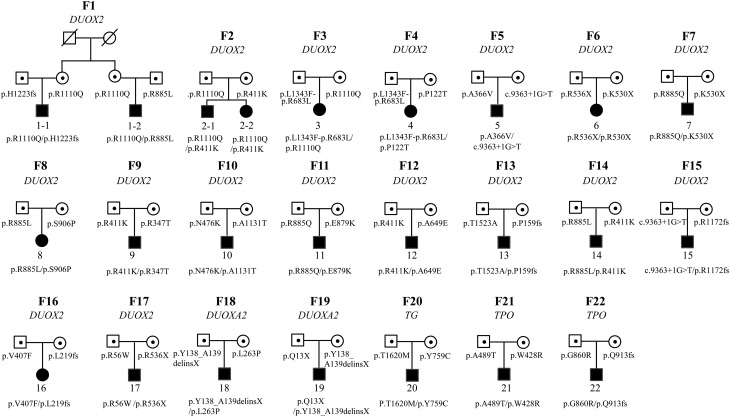
Twenty-two pedigrees with probands carried biallelic mutation. Seventeen pedigrees carried *DUOX2* mutations, 2 harbored *TPO* mutations, two had *DUOXA2* mutation, and one had *TG* mutation. For example, the mothers of proband 1 and 2 from family 1 were sisters who shared the same *DUOX2* mutation, which was transmitted to their children respectively. Another *DUOX2* mutation carried by proband 1 and 2 was inherited from their fathers respectively. The finding that individuals with biallelic mutations have congenital hypothyroidism (CH), and parents with heterozygous mutations are euthyroid indicates the CH caused by *DUOX2*, *DUOXA2*, *TG* and *TPO* is inherited in an autosomal recessive manner.

Among 22 family pedigrees with probands carrying biallelic mutations, 17 probands carried *DUOX2* mutations, 2 probands harbored *TPO* mutations, two had *DUOXA2* mutations and one carried *TG* mutations. However, hypothyroidism was not observed in their parents with heterozygous mutations of the four genes, revealing CH caused by mutations in *DUOX2*,* DUOXA2*,* TPO* and* TG* were inherited in an autosomal recessive manner (Fig. 4, Supplementary Table 3).

## Discussion

In this study, we analyzed the mutation patterns of 21 candidate genes in 110 Chinese patients with CH by next-generation sequencing. Of the 21 candidate genes, *TPO*, *SLC5A5*, *TG*, *TSHR*, *DUOX2*, *DUOXA2*, *SLC26A4*, *FOXE1*, *PAX8*, *NKX2-1*, *NKX2-5* and *IYD* were previously reported possible causative genes for CH, while the other nine genes: *DIO1*, *DIO2*, *THRA*, *THRB*, *DUOX1*, *DUOXA1*, *GNAS*, *SLC16A2* and* HHEX* were not well-established disease genes and were included based on their function associated with the production and transportation of thyroid hormone. So we further evaluated the 21 gene–disease pairs to assess the level of gene–disease association as described in the standard operating procedure document provided on the ClinGen website (25). We reached clinical validity classifications as ‘definitive’ in the following 13 gene–disease pairs with available published evidence:* SLC5A5*, *TG*, *TSHR*, *DUOX2*, *DUOXA2*, *SLC26A4*, *FOXE1*, *PAX8*, *NKX2-1*, *NKX2-5 IYD, THRA*, *THRB*-CH pairs and *GNAS*-CH pair were present to attain ‘strong’ classification, *DUOXA1*-CH pairs were assessed as ‘moderate’ classifications, the classifications for *DUOX1* was ‘limited’ and *HHEX*, *DIO1* and *DIO2* were classified as ‘no reported evidence’ associated with CH (Supplementary Table 4). It is worth noting that, although we classified ‘*DUOX1*-CH’ pair as ‘limited’ based on reported literatures, we found 11 patients harbored heterozygous mutations in *DUOX1* combined with or without other gene’s mutation(s) in our study. So we speculated that ‘*DUOX1*-CH’ pair may be upgraded to ‘moderate’ or ‘strong’ in the future. And for *HHEX* gene, one patient had a heterozygous mutation in our 110 patients although no variants have been reported in CH patients before, we supposed ‘*HHEX*-CH’ pair was likely to be upgraded to ‘limited’ or ‘moderate’ in consideration of its function along with more and more studies.

By next-generation sequencing, we identified 218 mutations in 15 of the genes, and they were distributed in 122 sites (29 novel sites and 93 previously reported sites) in 89 of the 110 patients. The most frequently mutated gene in these patients was *DUOX2*, followed by *TG*, *TPO*, *DUOXA1*,* DUOX1* and *TSHR*. Mutations were identified in 9 of 15 mutated genes involved in thyroid hormone synthesis (*DUOX2*, *TG*, *TPO*, *DUOXA1*, *DUOXA2*, *DUOX1*, *TSHR*, *SLC26A4* and *IYD*) in 87 (79.1%, 87/110) patients and 57 of these patients (51.82%, 57/110) carried biallelic mutations in *DUOX2*, *TG*, *TPO*, *TSHR, DUOXA2* and *DUOXA1*. In contrast, eight patients harbored heterozygous mutations in *NKX2-1*, *FOXE1*, *PAX8* or *HHEX*, which lead to thyroid dysgenesis. Moreover, in 110 CH we collected, 21 patients had goiter, 51 patients had normal-sized thyroid and 28 patients did not know their thyroid morphology and size. Only 10 patients had thyroid dysgenesis (including 2 patients with ectopic thyroid, 3 patients with thyroid athyreosis and 5 patients with thyroid hypoplasia), which was much less than thyroid dyshormonogenesis that led to normal size or goiter (26). In brief, these data suggest that thyroid dyshormonogenesis is a more common cause of CH than thyroid dysgenesis in the Chinese population.

Our data showed that 66 of the 110 patients with CH (60%, 66/110) carried *DUOX2* mutations, which is similar to the reported prevalence of this mutation (62.5%) in patients in Guangzhou, China (2). Forty-one of these patients (37.27%, 41/110) carried biallelic mutations in *DUOX2*. The *DUOX2* mutation appears to be the most common cause of permanent CH in East Asia, which is consistent with studies conducted in Korea (27) and Japan (28). However, some studies on the Caucasian population showed that *TPO* mutation is the most common cause of dyshormonogenesis (29, 30, 31). However, from our data, the mutation prevalence of *TPO* was 10.91% (12/110), which was much less than that of *DUOX2* (60%, 66/110), we supposed that the racial difference may well explain the difference of the main causative gene of dyshormonogenesis between East Asia and Western countries.

*DUOX2* protein is located at the apical membrane of thyrocytes and is involved in H_2_O_2_ generation. Because H_2_O_2_ is the limiting factor for thyroglobulin iodination when the iodide supply is normal (5), *DUOX2* mutations often cause thyroid dyshormonogenesis and are linked to CH associated with goiter or a thyroid gland of normal size in the presence of an iodide organification defect (32). Of the 41 CH patients with biallelic *DUOX2* mutations in our study, increased gland size was noted in 13, normal gland size/location was noted in 19 and 9 patients with unknown thyroid size. This finding is consistent with previous studies reporting that most patients with pathogenic *DUOX2* mutations have an increased or normal size thyroid gland (33). Previous study has reported that permanent CH has been associated with biallelic inactivating mutations in *DUOX2* while transient CH was described in patients bearing monoallelic mutations (34). In our study, *DUOX2* is the causative gene of these 41 patients with biallelic *DUOX2* mutations; however, *DUOX2* may not be the causative gene for other patients carrying monoallelic *DUOX2* mutation, other unknown causative genes needed to be further studied for these patients.

Thyroglobulin (Tg) is a glycoprotein homodimer produced predominantly by the thyroid gland. It acts as a substrate for the synthesis of thyroxine and triiodothyronine as well as the storage of the inactive forms of thyroid hormone and iodine. In our cohort, 18 patients (18/110, 16.36%) harbored TG mutations, the frequency of which was similar as that of 13.6% (9/66) by Fan *et al*. (35) and 15.18% (58/382) by Hu *et al*. (36). Of note, Hu *et al.* conducted a genetic screening of TG gene in a cohort of 382 Chinese CH patients and found the c.274+2T>G variant is the most common pathogenic variant with an allele frequency of 0.021, which is an ethnicity specific pathogenic variant with its uniquely high frequency in Chinese population and was only previously reported in two Taiwanese CH patients (37). However, both in our cohort of 110 Chinese patients with CH and 144 controls in-house, this variant was not found after excluding the sequencing reason. We speculated the difference may be explained by regional disparity or the limited sample size.

Although more than 21 genes are reported to be associated with CH, the inheritance modes of CH caused by mutations in these genes have been a matter of controversy. All forms of thyroid dyshormonogenesis were considered to be inherited in an autosomal recessive manner, with the exception of thyroid dyshormonogenesis caused by *DUOX2* mutations, which was thought to be transmitted in an autosomal dominant manner (38). However, increasing evidence supports the idea that CH caused by genes involved in organification defects, such as *DUOX2*, *TPO*, *DUOXA2* and *SLC26A4*, follows an autosomal recessive inheritance pattern (5, 17). To determine the inheritance mode of CH caused by thyroid dyshormonogenesis, we analyzed 22 family trios, in which the probands carried compound heterozygous mutations in genes involved in thyroid hormone synthesis (*DUOX2*, *DUOXA2*, *TPO* and *TG*). All parents with heterozygous mutations in these genes were euthyroid, suggesting that CH caused by mutations in these genes follows an autosomal recessive inheritance pattern (39, 40).

In contrast, thyroid dysgenesis was generally assumed to be a sporadic disease, it had been reported to have genetic background. So far, the inheritance mode in regard to thyroid dysgenesis has been controversial, some familial cases support the Mendelian inheritance, however, other modes of inheritance such as multigenetic, multifactorial as well as epigenetics could not be excluded (41, 42, 43, 44), and a significant discordance between monozygotic twins for thyroid dysgenesis was a strong argument against classic Mendelian inheritance (38). To determine the inheritance mode of CH caused by thyroid dysgenesis, we analyzed six family trios. Our results showed that parents with heterozygous mutations in *NKX2-1*, *PAX8* and *FOXE1* were euthyroid, suggesting that CH caused by mutations of *PAX8*, *NKX2-1* and *FOXE1* is not inherited in the classic autosomal dominant manner, which is inconsistent with previous reports (45). But whether these heterozygous mutations were loss of function or not, no other teams had already analyzed the mutation function as so far. Additional studies with large cohorts of patients with CH and functional studies are needed to confirm this finding.

Notably, the extraordinary finding in our study is the high frequency of mutations in *DUOX2* and the autosomal recessive inheritance of this gene, which is not consistent with genetic epidemiology of CH in Caucasian population, in which affected siblings are much less than 25%, whereas our data showed that the frequency affected siblings followed the classic Mendelian autosomal recessive inheritance (both siblings were affected in family 2). It is probably caused by that the frequency of mutation for *DUOX2* was rare in patients with CH in Western countries; however, most of the investigations about the genetic epidemiology of CH were based on Caucasian population.

Although we performed relative systematic and comprehensive screening for candidate genes of CH, some limitations should be considered in our study when reviewing our findings. First, this is a highly selected population and sample size is relatively small in our study. Second, we did not carry out functional studies of mutations in those 21 candidate genes. Therefore, further studies are still needed to expand the mutation spectrum of CH and relevant functional studies are needed to verify the function of variants, which may give a more profound insight to the etiology of CH.

In conclusion, this first systematic and comprehensive screening of 21 candidate genes in Chinese patients with CH showed that the most frequently mutated gene was *DUOX2*. Our result that 57/110 (51.82%) patients carried biallelic mutations in genes involved in thyroid hormone synthesis (*DUOX2*, *TPO*, *TG*, *DUOXA1*, *TSHR* or *DUOXA2*) suggests that in more than 50% of Chinese patients with CH, the disease is caused by thyroid dyshormonogenesis and is inherited in an autosomal recessive manner.

## Supplementary Material

Supporting Figure 1

Supporting Table 1

Supporting Table 2

Supporting Table 3

Supporting Table 4

## Declaration of interest

The authors declare that there is no conflict of interest that could be perceived as prejudicing the impartiality of this study.

## Funding

This work was supported in part by National Key R&D Program of China (2017YFC1001801), the National Natural Science Foundation of China (81670717, 31571296, 81430019, 81661168016, 81370888 and 31501015) and Shanghai Municipal Education Commission-Gaofeng Clinical Medicine Grant Support (20161318).

## Author contribution statement

H D S conceived and designed the project. F S, S X Z and H D S contribute to the project management. F S, Y Y W, X P Y, Y R M, M M Z, L Y, Q Y Z, C C G, W L and B H contributed to the next-generation sequencing. L L Z and Y R M took part in the statistical analysis. G Q G, W B Z, C Y Y, K Y S, G C and J X Z took part in the collection of clinical samples, extracted DNA and sample quality control. F S and H D S wrote the manuscript.
