# Supplementary figures and images for: The genetic characteristics of congenital hypothyroidism in China by comprehensive screening of 21 candidate genes
Source: Eur J Endocrinol. 2018 Mar 28;178(6):623–33. doi: 10.1530/EJE-17-1017 (PMC5958289; doi:10.1530/EJE-17-1017)

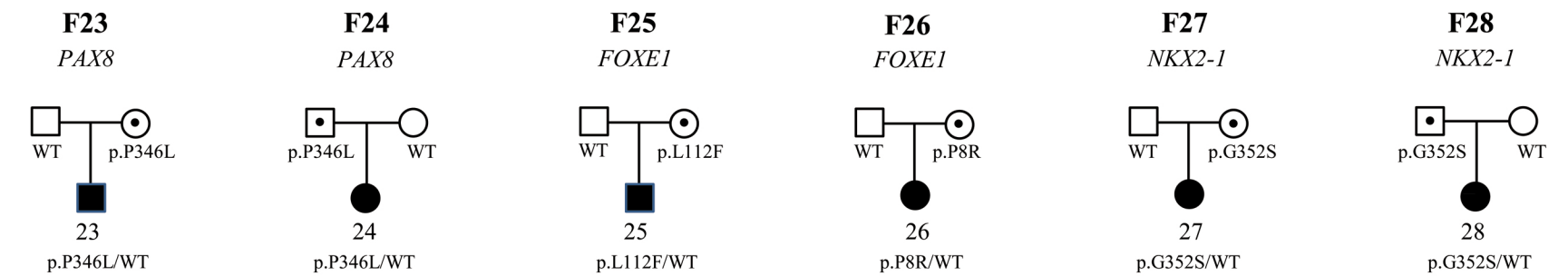

|              | 23 | 23-F | 23-M | 24   | 24-F | 24-M | 25   | 25-F | 25-M | 26 | 26-F | 26-M | 27   | 27-F | 27-M | 28   | 28-F | 28-M | Ref.values |
|--------------|----|------|------|------|------|------|------|------|------|----|------|------|------|------|------|------|------|------|------------|
| TSH (uIU/ml) | NA | 1.09 | 0.51 | 66.6 | 2.89 | 1.93 | >100 | 2.00 | 5.02 | NA | 3.53 | 3.87 | 53.5 | 3.66 | 1.62 | 9.62 | 0.44 | 0.76 | 0.34-5.6   |
| FT3 (pg/ml)  | NA | 3.29 | 3.08 | NA   | 3.39 | 3.51 | 2.81 | 3.98 | 3.27 | NA | 3.34 | 3.82 | 3.61 | 3.13 | 3.6  | 3.99 | 2.98 | 4.35 | 2.0-4.4    |
| FT4 (ng/dl)  | NA | 1.04 | 0.92 | NA   | 0.98 | 0.83 | 0.43 | 0.88 | 0.68 | NA | 0.87 | 0.94 | 1.01 | 1.03 | 1.18 | 1.12 | 1.14 | 1.03 | 0.93-1.7   |

Supplement: Supporting Figure 1 [file eje-178-623-s001.pdf]
