# Supplementary material for: The genetic characteristics of congenital hypothyroidism in China by comprehensive screening of 21 candidate genes
Source: Eur J Endocrinol. 2018 Mar 28;178(6):623–33. doi: 10.1530/EJE-17-1017 (PMC5958289; doi:10.1530/EJE-17-1017)
Supplement: Supporting Table 1 [file eje-178-623-t001.pdf]

Supplemental Table 1. The clinical and mutation information of 89 mutated CH patients in our study													
Case NO.	Gender	At diagnose					Mutation information						
			Age（d)	FT3（2.0-4.4）pg/ml	FT4（0.93-1.7）ng/dL	TSHR（0.27-4.2）uIU/mL	Mutated gene	Annotation	Mutation type	Mutation status	Inheritance	Reported or Unreported	Classify Sequence Variants according ACMG/AMP guideline
1	Female	goiter	30	2.72	0.41	˃ 100	*TSHR*	NM_003235:c.G1576A:p.A526T	Missense	Heterozygous	NA	reported	VUS
							DUOX2	NM_014080:c.C4027T:p.L1343F	Missense	Heterozygous	NA	reported	Pathogenic
							DUOX2	NM_014080:c.G2921A:p.R974H	Missense	Heterozygous	NA	reported	VUS
							DUOX2	NM_014080:c.G2794A:p.D932N	Missense	Heterozygous	NA	reported	VUS
							DUOX2	NM_014080:c.G2048T:p.R683L	Missense	Heterozygous	NA	reported	Pathogenic
2	Female	goiter	15	2.39	0.49	˃ 100	*DUOX2*	NM_014080:c.C4027T:p.L1343F	Missense	Heterozygous	NA	reported	Pathogenic
							DUOX2	NM_014080:c.A3902C:p.Q1301P	Missense	Homozygous	NA	unreported	Likely Pathogenic
							DUOX2	NM_014080:c.G2794A:p.D932N	Missense	Heterozygous	NA	reported	VUS
							DUOX2	NM_014080:c.G2048T:p.R683L	Missense	Heterozygous	NA	reported	Pathogenic

							DUOX1	NM_017434:c.G3920A:p.R1307Q	Missense	Heterozygous	NA	reported	VUS
3	Male	goiter	30	2.53	<0.40	>100	*SLC26A4*	NM_000441:c.919-2A>G	splicing	Heterozygous	NA	reported	Pathogenic
							DUOX2	NM_014080:c.C4027T:p.L1343F	Missense	Heterozygous	NA	reported	Pathogenic
							DUOX2	NM_014080:c.G2794A:p.D932N	Missense	Heterozygous	NA	reported	VUS
							DUOX2	NM_014080:c.G2048T:p.R683L	Missense	Heterozygous	NA	reported	Pathogenic
							DUOX2	NM_014080:c.A1588T:p.K530X	stopgain	Heterozygous	NA	reported	Pathogenic
4	Male	goiter	30	2.73	0.95	44.41	*TG*	NM_003235:c.C958T:p.R320C	Missense	Heterozygous	NA	reported	VUS
							DUOX2	NM_014080:c.G2635A:p.E879K	Missense	Heterozygous	NA	reported	Pathogenic
							DUOX2	NM_014080:c.G1868A:p.R623Q	Missense	Heterozygous	NA	reported	VUS
							DUOX2	NM_014080:c.G2921A:p.R974H	Missense	Heterozygous	NA	reported	VUS
5	Female	normal	30	2.19	0.28	100	*TSHR*	NM_003235:c.A1115C:p.N372T	Missense	Heterozygous	mother	unreported	VUS
							DUOX2	NM_014080:c.C4027T:p.L1343F	Missense	Heterozygous	father	reported	Pathogenic
							DUOX2	NM_014080:c.G2048T:p.R683L	Missense	Heterozygous	father	reported	Pathogenic
							DUOX2	NM_014080:c.C364A:p.P122T	Missense	Heterozygous	mother	reported	Likely Pathogenic
							DUOXA1	NM_001276268:c.C601T:p.H201Y	Missense	Heterozygous	mother	unreported	VUS
6	Female	norma	15	3.06	0.97	20.0	*DUOX2*	NM_014080:c.C4027	Missens	Heteroz	father	report	Pathog

		l				1		T:p.L1343F	e	ygous		ed	enic
							DUOX2	NM_014080:c.G3329A:p.R1110Q	Missense	Heterozygous	mother	reported	Pathogenic
							DUOX2	NM_014080:c.G2048T:p.R683L	Missense	Heterozygous	father	reported	Pathogenic
							DUOXA1	NM_001276268:c.C503T:p.T168M	Missense	Heterozygous	*De nove*	reported	Likely Pathogenic
7	Male	NA	30	NA	NA	NA	*PAX8*	NM_013952:c.C1037T:p.P346L	Missense	Heterozygous	mother	reported	Pathogenic
							DUOX2	NM_014080:c.T608C:p.L203P	Missense	Heterozygous	father	unreported	VUS
							DUOX2	NM_014080:c.612_620del:p.204_207del	nonframeshift deletion	Heterozygous	mother	reported	VUS
							DUOX2	NM_014080:c.602dupG:p.G201fs	frameshift insertion	Heterozygous	mother	reported	Pathogenic
8	Female	goiter	30	2.9	0.74	93.92	*DUOX2*	NM_014080:c.C4027T:p.L1343F	Missense	Heterozygous	NA	reported	Pathogenic
							DUOX2	NM_014080:c.G2654T:p.R885L	Missense	Heterozygous	NA	reported	Pathogenic
							DUOX2	NM_014080:c.G2048T:p.R683L	Missense	Heterozygous	NA	reported	Pathogenic
9	Female	NA	15	2.76	0.41	>100	*DUOX2*	NM_014080:c.C4027T:p.L1343F	Missense	Heterozygous	NA	reported	Pathogenic
							DUOX2	NM_014080:c.G2654A:p.R885Q	Missense	Heterozygous	NA	reported	Pathogenic
							DUOX2	NM_014080:c.G2048T:p.R683L	Missense	Heterozygous	NA	reported	Pathogenic
10	Male	normal	30	2.54	<0.40	>100	*TG*	NM_003235:c.C4859T:p.T1620M	Missense	Heterozygous	NA	reported	Likely benign

							TG	NM_003235:c.C7182G:p.I2394M	Missense	Heterozygous	NA	unreported	VUS
							DUOX2	NM_014080:c.G1310C:p.G437A	Missense	Heterozygous	NA	reported	VUS
							DUOX2	NM_014080:c.C505T:p.R169W	Missense;	Heterozygous	NA	reported	Pathogenic
11	Male	normal	23	3.91	0.57	130.53	*TG*	NM_003235:c.C3808T:p.R1270C	Missense	Heterozygous	NA	reported	VUS
							DUOX2	NM_014080:c.G3329A:p.R1110Q	Missense	Heterozygous	mother	reported	Pathogenic
							DUOX2	NM_014080:c.G2654T:p.R885L	Missense	Heterozygous	NA	reported	Pathogenic
							DUOXA1	NM_001276268:c.C503T:p.T168M	Missense	Heterozygous	mother	reported	Likely Pathogenic
							DUOX1	NM_017434:c.T3435A:p.S1145R	Missense	Heterozygous	NA	reported	VUS
12	Male	normal	13	NA	NA	150	*DUOX2*	NM_014080:c.G1232A:p.R411K	Missense	Heterozygous	father	reported	Likely Pathogenic
							TG	NM_003235:c.C7753T:p.R2585W	Missense	Heterozygous	father	reported	VUS
							DUOX2	NM_014080:c.G1040C:p.R347T	Missense	Heterozygous	mother	reported	VUS
13	Female	goiter	30	2.97	˂ 0.40	>100	*TPO*	NM_000547:c.G566A:p.R189Q	Missense	Heterozygous	NA	reported	VUS
							DUOX2	NM_014080:c.3693+1G>T	splicing	Heterozygous	NA	reported	Pathogenic
							DUOX2	NM_014080:c.C505T:p.R169W	Missense	Heterozygous	NA	reported	Pathogenic
14	Male	goiter	23	NA	NA	NA	*TPO*	NM_000547:c.T2291C:p.L764P	Missense	Homozygous	mother	unreported	VUS

							DUOX2	NM_014080:c.G3391A:p.A1131T	Missense	Heterozygous	mother	reported	Likely Pathogenic
							DUOX2	NM_014080:c.C1428A:p.N476K	Missense	Heterozygous	father	reported	VUS
15	Male	goiter	30	1.63	0.51	˃ 100	*DUOX2*	NM_014080:c.G3329A:p.R1110Q	Missense	Heterozygous	NA	reported	Pathogenic
							DUOX2	NM_014080:c.A1588T:p.K530X	stopgain	Heterozygous	NA	reported	Pathogenic
							DUOXA1	NM_001276268:c.C503T:p.T168M	Missense	Heterozygous	NA	reported	Likely Pathogenic
16	Female	NA	30	2.64	0.4	˃ 100	*DUOX2*	NM_014080:c.G3329A:p.R1110Q	Missense	Heterozygous	NA	reported	Pathogenic
							DUOX2	NM_014080:c.G2654T:p.R885L	Missense;	Heterozygous	NA	reported	Pathogenic
							DUOXA1	NM_001276268:c.C503T:p.T168M	Missense	Heterozygous	NA	reported	Likely Pathogenic
17	Male	normal	40	4.25	2.66	2.91	*DUOX2*	NM_014080:c.G3329A:p.R1110Q	Missense	Heterozygous	NA	reported	Pathogenic
							DUOXA1	NM_001276268:c.C503T:p.T168M	Missense	Heterozygous	mother	reported	Likely Pathogenic
							DUOX2	NM_014080:c.3667delC:p.H1223fs	frameshift deletion	Heterozygous	NA	reported	Pathogenic
18	Male	goiter	30	3.24	0.44	>100	*DUOX2*	NM_014080:c.G3329A:p.R1110Q	Missense	Heterozygous	NA	reported	Pathogenic
							DUOX2	NM_014080:c.A1588T:p.K530X	stopgain	Heterozygous	NA	reported	Pathogenic
							DUOXA1	NM_001276268:c.C5	Missens	Heteroz	NA	report	Likely

								03T:p.T168M	e	ygous		ed	Pathogenic
19	Female	normal	30	1.47	<0.40	>100	*DUOX2*	NM_014080:c.G3329A:p.R1110Q	Missense	Heterozygous	NA	reported	Pathogenic
							DUOX2	NM_014080:c.T959C:p.L320P	Missense	Heterozygous	NA	reported	Likely Pathogenic
							DUOXA1	NM_001276268:c.C503T:p.T168M	Missense	Heterozygous	NA	reported	Likely Pathogenic
20	Male	goiter	30	2.46	0.7	38.58	*TSHR*	NM_003235:c.A2146G:p.S716G	Missense	Heterozygous	NA	unreported	VUS
							DUOX2	NM_014080:c.G2654T:p.R885L	Missense	Heterozygous	NA	reported	Pathogenic
							DUOX2	NM_014080:c.A1588T:p.K530X	stopgain	Heterozygous	NA	reported	Pathogenic
21	Male	normal	50	NA	NA	16.87	*FOXE1*	NM_004473:c.C334T:p.L112F	Missense	Heterozygous	mother	reported	VUS
							DUOX2	NM_014080:c.G2654A:p.R885Q	Missense	Heterozygous	father	reported	Pathogenic
							DUOX2	NM_014080:c.G2635A:p.E879K	Missense	Heterozygous	mother	reported	Pathogenic
							DUOX1	NM_017434:c.T3435A:p.S1145R	Missense	Heterozygous	mother	reported	VUS
22	Male	normal	30	1.81	<0.4	>100	*DUOX2*	NM_014080:c.C1946A:p.A649E	Missense	Heterozygous	mother	reported	VUS
							DUOX1	NM_017434:c.T2957C:p.L986P	Missense	Heterozygous	mother	reported	VUS
							DUOX2	NM_014080:c.G1232A:p.R411K	Missense	Heterozygous	father	reported	Likely Pathogenic
23	Female	NA	30	NA	NA	NA	*DUOX2*	NM_014080:c.G2654T:p.R885L	Missense	Heterozygous	NA	reported	Pathogenic

							DUOX2	NM_014080:c.A1588T:p.K530X	stopgain	Heterozygous	NA	reported	Pathogenic
							GNAS	NM_016592:c.G838A:p.A280T	Missense	Heterozygous	NA	reported	VUS
24	Female	NA	30	NA	NA	NA	*DUOX2*	NM_014080:c.C3061T:p.R1021X	stopgain	Heterozygous	NA	reported	Pathogenic
							DUOX2	NM_014080:c.A1588T:p.K530X	stopgain	Heterozygous	NA	reported	Pathogenic
25	Male	normal	15	3.28	0.87	24.57	*DUOX2*	NM_014080:c.3693+1G>T	splicing	Heterozygous	NA	reported	Pathogenic
							DUOX2	NM_014080:c.T959C:p.L320P	Missense	Heterozygous	NA	reported	Likely Pathogenic
26	Male	goiter	30	3.37	0.45	˃ 100	*DUOX2*	NM_014080:c.3693+1G>T	splicing	Heterozygous	NA	reported	Pathogenic
							DUOX2	NM_014080:c.A1588T:p.K530X	stopgain	Heterozygous	NA	reported	Pathogenic
27	Female	normal	15	3.15	0.63	>100	*DUOX2*	NM_014080:c.C4027T:p.L1343F	Missense	Heterozygous	NA	reported	Pathogenic
							DUOX2	NM_014080:c.G2048T:p.R683L	Missense	Heterozygous	NA	reported	Pathogenic
28	Male	NA	28	NA	NA	NA	*DUOX2*	NM_014080:c.3693+1G>T	splicing	Heterozygous	NA	reported	Pathogenic
							DUOX2	NM_014080:c.1007_1009del:p.336_337del	nonframeshift deletion	Heterozygous	mother	unreported	VUS
29	Male	normal	19	NA	NA	NA	*DUOX2*	NM_014080:c.A4567G:p.T1523A	Missense	Heterozygous	father	reported	VUS
							DUOX2	NM_014080:c.477delC:p.P159fs	frameshift deletion	Heterozygous	mother	unreported	Pathogenic
30	Male	norma	40	3.66	1.3	8.88	*DUOX2*	NM_014080:c.3693+	splicing	Heteroz	mother	report	Pathog

		l						1G>T		ygous		ed	enic
							DUOX2	NM_014080:c.C1097T:p.A366V	Missense	Heterozygous	father	reported	VUS
31	Female	normal	46	4.65	1.04	17.58	*DUOX2*	NM_014080:c.C4027T:p.L1343F	Missense	Heterozygous	father	reported	Pathogenic
							DUOX2	NM_014080:c.G2048T:p.R683L	Missense	Heterozygous	father	reported	Pathogenic
32	Male	goiter	30	3.98	0.72	64.78	*DUOX2*	NM_014080:c.C1606T:p.R536X	stopgain	Heterozygous	mother	reported	Pathogenic
							DUOX2	NM_014080:c.C166T:p.R56W	Missense	Heterozygous	father	unreported	VUS
33	Male	normal	20	1.44	0.25	>150	*DUOX2*	NM_014080:c.G1232A:p.R411K	Missense	Heterozygous	mother	reported	Likely Pathogenic
							DUOX2	NM_014080:c.G2654T:p.R885L	Missense	Heterozygous	father	reported	Pathogenic
34	Male	goiter	30	NA	NA	NA	*DUOX2*	NM_014080:c.G4318A:p.D1440N	Missense	Heterozygous	NA	reported	VUS
							DUOX2	NM_014080:c.C1873T:p.R625X	stopgain	Heterozygous	NA	reported	Pathogenic
35	Male	NA	30	3.41	0.66	39.55	*TG*	NM_003235:c.C2222T:p.T741M	Missense	Heterozygous	NA	reported	Likely benign
							TG	NM_003235:c.A7847T:p.N2616I	Missense	Heterozygous	NA	reported	VUS
							DUOX2	NM_014080:c.G3329A:p.R1110Q	Missense	Heterozygous	NA	reported	Pathogenic
							DUOXA1	NM_001276268:c.C503T:p.T168M	Missense	Heterozygous	NA	reported	Likely Pathogenic
							DUOXA1	NM_001276268:c.C166T:p.R56W	Missense	Heterozygous	NA	reported	VUS
36	Male	norma	30	4.07	0.52	>100	*TG*	NM_003235:c.C1333	stopgain	Heteroz	NA	report	Pathog

		l						T:p.R445X		ygous		ed	enic
							TG	NM_003235:c.C2281T:p.P761S	Missense	Heterozygous	NA	reported	VUS
							DUOX2	NM_014080:c.G2654T:p.R885L	Missense	Heterozygous	NA	reported	Pathogenic
							TPO	NM_000547:c.2268dupT:p.C756fs	frameshift insertion	Heterozygous	NA	reported	Pathogenic
							TG	NM_003235:c.5409delA:p.T1803fs	frameshift deletion	Heterozygous	NA	unreported	Pathogenic
37	Male	athyreosis	40	NA	NA	150	*SLC26A4*	NM_000441:c.C147G:p.S49R	Missense	Heterozygous	mother	reported	VUS
							TG	NM_003235:c.A2276G:p.Y759C	Missense	Heterozygous	mother	reported	VUS
							TG	NM_003235:c.C4859T:p.T1620M	Missense	Heterozygous	father	reported	Likely benign
38	Female	normal	30	4.26	0.51	66.74	*TG*	NM_003235:c.C705A:p.C235X	stopgain	Heterozygous	NA	unreported	Pathogenic
							TG	NM_003235:c.1348delT:p.S450fs	frameshift deletion	Heterozygous	NA	reported	Pathogenic
39	Male	NA	30	NA	NA	NA	*TSHR*	NM_003235:c.T647C:p.I216T	Missense	Heterozygous	NA	reported	VUS
							TSHR	NM_003235:c.G823A:p.A275T	Missense	Heterozygous	NA	reported	VUS
							DUOX2	NM_014080:c.A1087G:p.S363G	Missense	Heterozygous	NA	unreported	VUS
40	Male	NA	30	NA	NA	NA	*DUOX2*	NM_014080:c.G3632A:p.R1211H	Missense	Heterozygous	NA	reported	Likely Pathogenic
							TSHR	NM_003235:c.G1349	Missens	Heteroz	NA	report	Pathog

								A:p.R450H	e	ygous		ed	enic
							TSHR	NM_003235:c.C1582A:p.R528S	Missense	Heterozygous	NA	reported	VUS
41	Male	goiter	45	2.38	0.52	19.07	*TSHR*	NM_003235:c.G394C:p.G132R	Missense	Heterozygous	NA	reported	Pathogenic
							TSHR	NM_003235:c.G1349A:p.R450H	Missense	Heterozygous	NA	reported	Pathogenic
42	Male	normal	40	NA	NA	NA	*DUOXA2*	NM_207581:c.T788C:p.L263P	Missense	Heterozygous	mother	unreported	VUS
							DUOXA2	NM_207581:c.413dupA:p.Y138_A139delinsX	stopgain	Heterozygous	father	reported	Pathogenic
43	Male	normal	19	NA	NA	>150	*TPO*	NM_000547:c.T1282A:p.W428R	Missense	Heterozygous	mother	unreported	VUS
							TPO	NM_000547:c.G1465A:p.A489T	Missense	Heterozygous	father	reported	VUS
							DUOX2	NM_014080:c.3478_3480del:p.1160_1160del	nonframeshift deletion	Heterozygous	mother	reported	VUS
44	Female	thyroaplasia	30	3.61	1.01	53.51	*NKX2-1*	NM_001079668:c.G1054A:p.G352S	Missense	Heterozygous	mother	reported	VUS
							TSHR	NM_003235:c.A2252G:p.K751R	Missense	Heterozygous	mother	reported	VUS
							DUOX2	NM_014080:c.C1428A:p.N476K	Missense	Heterozygous	father	reported	VUS
45	Female	normal	15	NA	NA	NA	*DUOX2*	NM_014080:c.G3329A:p.R1110Q	Missense	Heterozygous	NA	reported	Pathogenic
							DUOXA1	NM_001276268:c.C503T:p.T168M	Missense	Heterozygous	mother	reported	Likely Pathogenic
							FOXE1	NM_004473:c.C23G:	Missens	Heteroz	mother	report	VUS

								p.P8R	e	ygous		ed	
46	Female	NA	15	1.79	<0.40	>100	*TG*	NM_003235:c.A1330G:p.I444V	Missense	Heterozygous	NA	reported	VUS
							DUOX2	NM_014080:c.T3395A:p.M1132K	Missense	Heterozygous	NA	reported	Pathogenic
47	Male	NA	30	3.11	0.54	71.66	*TG*	NM_003235:c.C4859T:p.T1620M	Missense	Heterozygous	NA	reported	Likely benign
							DUOX2	NM_014080:c.C534G:p.W178C	Missense	Heterozygous	NA	unreported	Likely Pathogenic
							DUOX2	NM_014080:c.A1588T:p.K530X	stopgain	Heterozygous	NA	reported	Pathogenic
48	Female	NA	30	3.66	1.01	32.4965	*DUOX2*	NM_014080:c.G3329A:p.R1110Q	Missense	Heterozygous	NA	reported	Pathogenic
							DUOXA1	NM_001276268:c.C503T:p.T168M	Missense	Heterozygous	NA	reported	Likely Pathogenic
							TPO	NM_000547:c.2268dupT:p.C756fs	frameshift insertion	Heterozygous	NA	reported	Pathogenic
49	Female	NA	30	NA	NA	NA	*TPO*	NM_000547:c.G1327C:p.A443P	Missense	Homozygous	NA	reported	Likely Pathogenic
							DUOX2	NM_014080:c.G2635A:p.E879K	Missense	Heterozygous	NA	reported	Pathogenic
							DUOX1	NM_017434:c.C415A:p.R139S	Missense	Heterozygous	NA	reported	Likely benign
50	Female	goiter	30	NA	NA	NA	*TPO*	NM_000547:c.G1327C:p.A443P	Missense	Homozygous	NA	reported	Likely Pathogenic
							DUOX2	NM_014080:c.A1588T:p.K530X	stopgain	Heterozygous	NA	reported	Pathogenic

51	Male	normal	30	NA	NA	9.4	*DUOX2*	NM_014080:c.G3632A:p.R1211H	Missense	Heterozygous	mother	reported	Likely Pathogenic
							TSHR	NM_003235:c.G326A:p.R109Q	Missense	Heterozygous	mother	reported	VUS
52	Male	normal	30	3.991	1.12	9.622	*NKX2-1*	NM_001079668:c.G1054A:p.G352S	Missense	Heterozygous	father	reported	VUS
							DUOX2	NM_014080:c.3693+1G>T	splicing	Heterozygous	father	reported	Pathogenic
							DUOX2	NM_014080:c.3219_3234del:p.A1073fs	frameshift-near-splice	Heterozygous	mother	unreported	Pathogenic
53	Female	goiter	24	NA	NA	NA	*DUOX1*	NM_017434:c.C3236A:p.T1079N	Missense	Heterozygous	mother	unreported	VUS
							DUOX2	NM_014080:c.1871delG:p.G624fs	frameshift deletion	Heterozygous	mother	reported	Pathogenic
54	Male	NA	30	2.59	0.46	>100	*DUOX2*	NM_014080:c.C4000T:p.R1334W	Missense	Heterozygous	NA	reported	Likely Pathogenic
55	Male	normal	20	NA	NA	NA	*DUOX2*	NM_014080:c.A1588T:p.K530X	stopgain	Heterozygous	father	reported	Pathogenic
56	Female	normal	21	3.89	0.77	54.59	*DUOX2*	NM_014080:c.G3616A:p.A1206T	Missense	Homozygous	mother,father	reported	Pathogenic
57	Male	normal	30	NA	NA	>150	*DUOX2*	NM_014080:c.G1462A:p.G488R	Missense	Heterozygous	father	reported	Likely Pathogenic
58	Male	normal	37	NA	NA	22.66	*TPO*	NM_000547:c.G2578A:p.G860R	Missense	Heterozygous	father	reported	VUS
							TPO	XM_005264700.1:c.2735dupT:p.Q913fs	frameshift insertion	Heterozygous	mother	reported	Likely Pathogenic

							TG	NM_003235:c.G3023A:p.R1008H	Missense	Heterozygous	father	reported	VUS
59	Female	normal	30	NA	NA	66.64	*PAX8*	NM_013952:c.C1037T:p.P346L	Missense	Heterozygous	father	reported	Pathogenic
							DUOXA2	NM_207581:c.C488T:p.P163L	Missense	Heterozygous	father	reported	VUS
							GNAS	NM_016592:c.C205A:p.H69N	Missense	Heterozygous	father	reported	VUS
							TG	NM_003235:c.C3067T:p.R1023W	Missense	Heterozygous	father	reported	VUS
60	Female	normal	15	NA	NA	>150	*SLC26A4*	NM_000441:c.A1087C:p.I363L	Missense	Heterozygous	mother	unreported	VUS
							TG	NM_003235:c.G5486C:p.R1829P	Missense	Heterozygous	father	reported	VUS
61	Female	normal	15	NA	NA	95	*TG*	NM_003235:c.A7847T:p.N2616I	Missense	Heterozygous	father	reported	VUS
62	Female	thyroaplasia	15	2.56	0.61	150	*TPO*	NM_000547:c.C2647T:p.P883S	Missense	Heterozygous	NA	reported	VUS
63	Male	goiter	15	1.27	<0.40	>100	*HHEX*	NM_002729:c.A199G:p.T67A	Missense	Heterozygous	NA	unreported	VUS
							TPO	NM_000547:c.2268dupT:p.C756fs	frameshift insertion	Homozygous	NA	reported	Pathogenic
64	Female	goiter	30	1.47	‹ 0.4	› 100	*TPO*	NM_000547:c.2268dupT:p.C756fs	frameshift insertion	Homozygous	NA	reported	Pathogenic
65	Male	goiter	15	1.89	<0.40	56.05	*TPO*	NM_000547:c.G1327C:p.A443P	Missense	Heterozygous	NA	reported	Likely Pathogenic
66	Female	normal	30	4.07	1.65	7.37	*DUOX1*	NM_017434:c.A580G:p.R194G	Missense	Heterozygous	NA	reported	Likely benign

							DUOXA2	NM_207581:c.413dupA:p.Y138_A139delinsX	stopgain	Homozygous	NA	reported	Pathogenic
67	Female	normal	30	NA	NA	NA	*DUOX1*	NM_017434:c.A1707T:p.R569S	Missense	Heterozygous	mother	reported	Likely benign
							DUOX2	NM_014080:c.C1219A:p.V407F	Missense	Heterozygous	father	unreported	VUS
							DUOX2	NM_014080:c.655delC:p.L219fs	frameshift deletion	Heterozygous	mother	unreported	Pathogenic
68	Female	thyroaplasia	60	3.68	0.84	>150	*DUOX1*	NM_017434.3:c.3524+1 - c.3524+4del	splicing	Heterozygous	NA	reported	Pathogenic
69	Female	NA	30	NA	NA	NA	*DUOX1*	NM_017434:c.C1117A:p.P373T	Missense	Heterozygous	NA	reported	VUS
70	Male	ectopy	30	3.14	1.28	43	*SLC26A4*	NM_000441:c.A2168G:p.H723R	Missense	Heterozygous	NA	reported	VUS
71	Male	normal	30	1.32	0.35	150.2	*PAX8*	NM_013952:c.C1037T:p.P346L	Missense	Heterozygous	NA	reported	Pathogenic
72	Female	normal	20	NA	NA	135.896	*GNAS*	NM_016592:c.G334A:p.E112K	Missense	Heterozygous	mother	reported	VUS
73	Male	normal	40	NA	NA	NA	*DUOX2*	NM_014080:c.G2635A:p.E879K	Missense	Heterozygous	NA	reported	pathogenic
74	Female	NA	NA	NA	NA	NA	*DUOX1*	NM_017434:c.G149A:p.R50Q	Missense	Heterozygous	NA	reported	VUS
							IYD	NM_001164694:c.A706G:p.M236V	Missense	Heterozygous	NA	unreported	VUS
75	Female	normal	20	2.47	0.61985	>100	*DUOXA2*	NM_207581:c.C37T:p.Q13X	stopgain	Heterozygous	father	unreported	Pathogenic
							DUOXA2	NM_207581:c.413dupA:p.Y138_A139delinsX	stopgain	Heterozygous	mother	reported	Pathogenic

76	Male	normal	30	3.2565	0.56287	>100	*DUOX2*	NM_014080:c.C3631T:p.R1211C	Missense	Heterozygous	NA	reported	VUS
							TG	NM_003235:c.C2183A:p.T728K	Missense	Heterozygous	NA	unreported	VUS
77	Female	normal	NA	4.4395	0.53284	37.19	*DUOX2*	NM_014080:c.G4093A:p.G1365R	Missense	Heterozygous	NA	unreported	VUS
78	Female	normal	6	2.028	0.38346	>100	*DUOX2*	NM_014080:c.T2716C:p.S906P	Missense	Heterozygous	father	reported	VUS
							DUOX2	NM_014080:c.G2654T:p.R885L	Missense	Heterozygous	mother	reported	VUS
79	Male	athyreosis	30	2.6065	1.0241	75	*TG*	NM_003235:c.T8296A:p.Y2766N	Missense	Heterozygous	NA	reported	VUS
80	Female	normal	30	3.276	0.84777	46.45	*DUOXA2*	NM_207581:c.T788C:p.L263P	Missense	Heterozygous	NA	unreported	VUS
	Female	normal	5	3.107	0.17402	>100	*DUOX2*	NM_014080：c.G3632A:p.R1211H	Missense	Heterozygous	NA	reported	Likely pathogenic
82	Male	normal	12	2.4895	0.91707	>100	*TG*	NM_003235:c.G7411T:p.A2471S	Missense	Heterozygous	NA	unreported	VUS
83	Female	NA	13	1.9825	0.22561	>100	*DUOX2*	NM_014080:c.3478_3480del:p.1160_1160del	nonframeshift deletion	Heterozygous	NA	reported	VUS
							THRA	NM_001190918:c.A508G:p.I170V	Missense	Heterozygous	NA	unreported	Likely benign
84	Female	ectopia	16	2.0345	0.63756	>100	*DUOX2*	NM_014080c.1300_1320del:p.434_440del	nonframeshift deletion	Heterozygous	NA	unreported	VUS
85	Female	NA	15	1.5665	0.26642	>100	*DUOX2*	NM_014080:c.2314_2316del:p.772_772del	nonframeshift deletion	Heterozygous	NA	reported	VUS
86	Male	normal	30	2.418	0.49819	69.64	*DUOX2*	NM_014080:c.C1606T:p.R536X	stopgain	Heterozygous	father	reported	pathogenic

							DUOX2	NM_014080:c.A1588T:p.K530X	stopgain	Heterozygous	mother	reported	pathogenic
							TG	NM_003235:c.C1175T:p.P392L	Missense	Heterozygous	NA	reported	VUS
87	Female	normal	14	1.4755	0.20405	>100	*DUOX2*	NM_014080:c.G2654A:p.R885Q	Missense	Heterozygous	father	reported	pathogenic
							DUOX2	NM_014080:c.A1588T:p.K530X;	stopgain	Heterozygous	mother	reported	pathogenic
88	Male	normal	20	2.197	0.28952	91.11	*DUOX2*	NM_014080:c.G3329A:p.R1110Q	Missense	Heterozygous	father	reported	pathogenic
							DUOX2	NM_014080:c.G1232A:p.R411K	Missense	Heterozygous	mother	reported	Likely pathogenic
							DUOXA1	NM_001276265:c.C503T:p.T168M	Missense	Heterozygous	NA	reported	Likely pathogenic
89	Female	NA	NA	NA	NA	NA	*DUOX2*	NM_014080:c.G3329A:p.R1110Q	Missense	Heterozygous	father	reported	pathogenic
							DUOX2	NM_014080:c.G1232A:p.R411K	Missense	Heterozygous	mother	reported	Likely pathogenic
							DUOXA1	NM_001276265:c.C503T:p.T168M	Missense	Heterozygous	NA	reported	Likely pathogenic

The thyroid hormone levels at screening of 20 patients were absolutely absent,while their parents nuncupated their children had CH by neonatal screening.d,days;NA,not available;VUS,variants uncertain significance.
