# Supplementary material for: The genetic characteristics of congenital hypothyroidism in China by comprehensive screening of 21 candidate genes
Source: Eur J Endocrinol. 2018 Mar 28;178(6):623–33. doi: 10.1530/EJE-17-1017 (PMC5958289; doi:10.1530/EJE-17-1017)
Supplement: Supporting Table 2 [file eje-178-623-t002.pdf]

Supplemental Table 2. The basic information of 122 identified mutation sites.

Gene	Annotation	NO. of mutation sample	Mutation type	Reported or Unreported	Classify Sequence Variants according ACMG/AMP guideline	The frequency observed in our study	The frequency observed in public databases											the fucntion of mutation	Ref / Database
							esp6500siv2_all	1000g2015aug_all	1000g2015aug_eas	ExAC_ALL	ExAC_AFR	ExAC_AMR	ExAC_EAS	ExAC_FIN	ExAC_NFE	ExAC_OTH	ExAC_SAS		
DUOX2	NM_014080:c.G3329A:p.R1110Q	12	missense	reported	pathogenic	0.1091	.	.	.	2.48E-05	0.0003	0	0	0	0	0	0	loss of function	Jin HY,et, al.Horm Res Paediatr. 2014
DUOX2	NM_014080:c.A1588T:p.K530X	12	stopgain	reported	pathogenic	0.1091	0.0003	0.0001997	.	9.92E-05	0.0009	0	0.0003	0	0	0	0		
DUOX2	NM_014080:c.C4027T:p.L1343F	9	missense	reported	pathogenic	0.0818	.	.	.	7.42E-05	0	0	0.0003	0	9.00E-05	0	0		
DUOX2	NM_014080:c.	9	misse	rep	pat	0.0	.	.	.	.	.	.	.	.	.	.	.		

	G2048T:p.R683L		nse	orted	hogenic	818													
DUOX2	NM_014080:c.G2654T:p.R885L	7	missense	reported	pathogenic	0.0636	.	.	.	.	.	.	.	.	.	.	.		
DUOX2	NM_014080:c.3693+1G>T	6	splicing	reported	pathogenic	0.0545	.	0.0001997	0.001	0.0003	0	0.0002	0.0034	0	3.00E-05	0	0.0001		
DUOX2	NM_014080:c.G1232A:p.R411K	5	missense	reported	Likely pathogenic	0.0455	0.0002	0.0001997	.	0.0002	9.66E-05	8.68E-05	0.001	0	0.0001	0	0.0002		
DUOX2	NM_014080:c.G2654A:p.R885Q	4	missense	reported	pathogenic	0.0364	.	.	.	2.48E-05	0	0.0002	0	0	1.50E-05	0	0	loss of function	Jin HY,et, al.Horm Res Paediatr. 2014
DUOX2	NM_014080:c.G2635A:p.E879K	4	missense	reported	pathogenic	0.0364	.	0.0013978	0.0069	0.0005	0	0.0002	0.0067	0	0	0	0	loss of function	Jin HY,et, al.Horm Res Paediatr. 2014
DUOX2	NM_014080:c.G3632A:p.R1211H	3	missense	reported	Likely pathogenic	0.0273	7.70E-05	0.0001997	.	1.65E-05	0	0	0	0	3.00E-05	0	0		
DUOX2	NM_014080:c.G2794A:p.D932N	3	missense	reported	VUS	0.0273	.	0.0001997	.	2.49E-05	0	0	0	0	3.33E-05	0	6.08E-05		
DUOX2	NM_014080:c.	2	misse	rep	VUS	0.0	0.00	.	.	7.4	0	0	0.0	0	9.0	0	0		

	G2921A:p.R974H		nse	orted		182	02			2E-05			003		0E-05				
DUOX2	NM_014080:c.C1606T:p.R536X	2	stopgain	reported	pathogenic	0.0182	.	.	.	.	.	.	.	.	.	.	.		
DUOX2	NM_014080:c.C1428A:p.N476K	2	missense	reported	VUS	0.0182	0.0003	0.0007987	0.002	0.0003	0.0002	8.64E-05	0.0003	0	0.0003	0	0.0007		
DUOX2	NM_014080:c.T959C:p.L320P	2	missense	reported	Likely pathogenic	0.0182	.	.	.	4.14E-05	0	0.0002	0.0001	0	3.01E-05	0	0		
DUOX2	NM_014080:c.C505T:p.R169W	2	missense	reported	pathogenic	0.0182	.	.	.	0.0002	0.0002	0	0.0024	0	3.32E-05	0	0		
DUOX2	NM_014080:c.3478_3480del:p.1160_1160del	2	nonframeshift deletion	reported	VUS	0.0182	.	.	.	.	.	.	.	.	.	.	.	loss of function	Yoshizawa-Ogasawara A,et al.J Pediatr Endocrinol Metab. 2016
DUOX2	NM_014080:c.C4000T:p.R1334W	1	missense	reported	Likely pathogenic	0.0091	.	0.0001997	0.001	8.24E-06	0	0	0.0001	0	0	0	0	loss of function	Jin HY,et, al.Horm Res Paediatr. 2014
DUOX2	NM_014080:c.A3902C:p.Q1301P	1	missense	unreported	Likely pathogenic	0.0091	.	.	.	3.34E-05	0	0	0.0005	0	0	0	0		
DUOX2	NM_014080:c.	1	fram	rep	pat	0.0	.	.	.	.	.	.	.	.	.	.	.		

	3667delC:p.H1223fs		eshift deletion	orted	hogenic	091													
DUOX2	NM_014080:c.G3616A:p.A1206T	1	missense	reported	pathogenic	0.0091	.	.	.	7.41E-05	0	8.64E-05	0	0	1.50E-05	0	0.0004		
DUOX2	NM_014080:c.T3395A:p.M1132K	1	missense	reported	pathogenic	0.0091	.	.	.	.	.	.	.	.	.	.	.		
DUOX2	NM_014080:c.G3391A:p.A1131T	1	missense	reported	Likely pathogenic	0.0091	.	.	.	.	.	.	.	.	.	.	.		
DUOX2	NM_014080:c.C3061T:p.R1021X	1	stopgain	reported	pathogenic	0.0091	.	.	.	.	.	.	.	.	.	.	.		
DUOX2	NM_014080:c.1871delG:p.G624fs	1	frameshift deletion	reported	pathogenic	0.0091	7.70E-05	0.0015974	0.0079	0.0005	0.0003	8.65E-05	0.006	0	1.50E-05	0.011	6.06E-05		
DUOX2	NM_014080:c.G1868A:p.R623Q	1	missense	reported	VUS	0.0091	.	.	.	.	.	.	.	.	.	.	.		
DUOX2	NM_014080:c.G1310C:p.G437A	1	missense	reported	VUS	0.0091	.	.	.	.	.	.	.	.	.	.	.		
DUOX2	NM_014080:c.C1097T:p.A366V	1	missense	reported	VUS	0.0091	.	.	.	1.65E-05	0	0	0	0	0	0	0.0001		
DUOX2	NM_014080:c.	1	misse	unr	VUS	0.0	.	0.000	0.001	8.2	0	0	0.0	0	0	0	0		

	A1087G:p.S363G		nse	eported		091		1997		5E-06			001						
DUOX2	NM_014080:c.G1040C:p.R347T	1	missense	reported	VUS	0.0091	.	.	.	8.24E-06	0	8.64E-05	0	0	0	0	0		
DUOX2	NM_014080:c.1007_1009del:p.336_337del	1	nonframeshift deletion	unreported	VUS	0.0091	7.70E-05	0.0001997	.	4.94E-05	0.0003	0	0	0	4.50E-05	0	0		
DUOX2	NM_014080:c.612_620del:p.204_207del	1	nonframeshift deletion	reported	VUS	0.0091	.	0.0021965	0.0109	0.0011	9.61E-05	0	0.0148	0	0	0	0.0001		
DUOX2	NM_014080:c.T608C:p.L203P	1	missense	unreported	VUS	0.0091	.	.	.	.	.	.	.	.	.	.	.		
DUOX2	NM_014080:c.602dupG:p.G201fs	1	frameshift insertion	reported	pathogenic	0.0091	.	.	.	.	.	.	.	.	.	.	.		
DUOX2	NM_014080:c.477delC:p.P159fs	1	frameshift deletion	unreported	pathogenic	0.0091	0.0002	0.0013978	0.0069	0.0005	0.0004	0	0.0055	0	3.23E-05	0	0.0001		
DUOX2	NM_014080:c.C364A:p.P122T	1	missense	reported	Likely pathog	0.0091	.	0.0023962	0.0119	0.0016	0	0	0.0206	0	7.50E-05	0.0055	0.0002		

					enic														
DUOX2	NM_014080:c.C166T:p.R56W	1	missense	unreported	VUS	0.0091	.	.	.	.	.	.	.	.	.	.	.		
DUOX2	NM_014080:c.C1946A:p.A649E	1	missense	reported	VUS	0.0091	.	0.0001997	0.001	3.30E-05	0	0	0.0005	0	0	0	0		
DUOX2	NM_014080:c.G4318A:p.D1440N	1	missense	reported	VUS	0.0091	.	.	.	.	.	.	.	.	.	.	.		
DUOX2	NM_014080:c.C1873T:p.R625X	1	missense	reported	pathogenic	0.0091	.	.	.	2.47E-05	0	0	0.0003	0	0	0	0		
DUOX2	NM_014080:c.G1462A:p.G488R	1	missense	reported	Likely pathogenic	0.0091	.	.	.	0	0	0	0	0	0	0	0		
DUOX2	NM_014080:c.655delC:p.L219fs	1	frameshift deletion	unreported	pathogenic	0.0091	.	.	.	0.0003	0	0	0.0043	0	0	0	0		
DUOX2	NM_014080:c.C534G:p.W178C	1	missense	unreported	Likely pathogenic	0.0091	.	.	.	.	.	.	.	.	.	.	.		
DUOX2	NM_014080:c.3219_3234del:p.A1073fs	1	frameshift-	unreport	pathogenic	0.0091	.	0.0003994	0.002	0.0001	0	0	0.0017	0	0	0	0		

			near-splice	ed															
DUOX2	NM_014080:c.C1219A:p.V407F	1	missense	unreported	VUS	0.0091	.	0.0015974	0.004	0.001	0.0002	0.0005	0.0103	0	0.0002	0	0.0004		
DUOX2	NM_014080:c.G4093A:p.G1365R	1	missense	unreported	VUS	0.0091	.	0.000599	0.003	0.0003	0	0	0.0039	0	0	0	0		
DUOX2	NM_014080:c.C3631T:p.R1211C	1	missense	reported	VUS	0.0091	.	.	.	.	.	.	.	.	.	.	.		
DUOX2	NM_014080:c.T2716C:p.S906P	1	missense	reported	VUS	0.0091	.	.	.	.	.	.	.	.	.	.	.		
DUOX2	NM_014080:c.1300_1320del:p.434_440del	1	nonframeshift deletion	unreported	VUS	0.0091	.	0.0001997	0.001	0.0002	0	0	0.0023	0	0	0	0		
DUOX2	NM_014080:c.2314_2316del:p.772_772del	1	nonframeshift deletion	reported	VUS	0.0091	.	0.0009984	0.005	0.0004	0	0	0.005	0	0	0.0011	0		
DUOX2	NM_014080:c.A4567G:p.T1523A	1	missense	reported	VUS	0.0091	.	.	.	4.12E-05	0	0	0.0005	0	1.50E-05	0	0		
TG	NM_003235:c.C4859T:p.T1620M	3	missense	reported	Likely beni	0.0273	0.0002	0.0009984	0.005	0.0005	0.0003	0	0.0068	0	0	0.0011	0.0002		

					gn														
TG	NM_003235:c.A7847T:p.N2616I	2	missense	reported	VUS	0.0182	0.0002	.	.	5.77E-05	9.62E-05	0	0.0005	0	3.00E-05	0	0		
TG	NM_003235:c.C705A:p.C235X	1	stopgain	unreported	pathogenic	0.0091	.	0.0003994	0.002	6.63E-05	0	0	0.0009	0	0	0	0		
TG	NM_003235:c.C958T:p.R320C	1	missense	reported	VUS	0.0091	.	.	.	8.36E-06	0	0	0.0001	0	0	0	0		
TG	NM_003235:c.A1330G:p.I444V	1	missense	reported	VUS	0.0091	.	.	.	.	.	.	.	.	.	.	.		
TG	NM_003235:c.C1333T:p.R445X	1	stopgain	reported	pathogenic	0.0091	.	.	.	.	.	.	.	.	.	.	.		
TG	NM_003235:c.1348delT:p.S450fs	1	frameshift deletion	reported	pathogenic	0.0091	.	.	.	.	.	.	.	.	.	.	.		
TG	NM_003235:c.C2222T:p.T741M	1	missense	reported	Likely benign	0.0091	.	.	.	.	.	.	.	.	.	.	.		
TG	NM_003235:c.A2276G:p.Y759C	1	missense	reported	VUS	0.0091	.	.	.	.	.	.	.	.	.	.	.		
TG	NM_003235:c.C2281T:p.P761S	1	missense	reported	VUS	0.0091	0.0024	0.0035943	0.002	0.0039	0.0017	0.005	0.0036	0.0029	0.0031	0.0055	0.0082		

TG	NM_003235:c.G3023A:p.R1008H	1	missense	reported	VUS	0.0091	.	0.000599	0.003	0.0001	0	0	0.0012	0	3.01E-05	0	0		
TG	NM_003235:c.C3067T:p.R1023W	1	missense	reported	VUS	0.0091	.	.	.	.	.	.	.	.	.	.	.		
TG	NM_003235:c.C3808T:p.R1270C	1	missense	reported	VUS	0.0091	.	0.0001997	0.001	8.24E-06	0	0	0.0001	0	0	0	0		
TG	NM_003235:c.5409delA:p.T1803fs	1	frameshift deletion	unreported	pathogenic	0.0091	.	0.0007987	0.004	4.12E-05	0	0	0.0006	0	0	0	0		
TG	NM_003235:c.G5486C:p.R1829P	1	missense	reported	VUS	0.0091	.	.	.	.	.	.	.	.	.	.	.		
TG	NM_003235:c.C7182G:p.I2394M	1	missense	unreported	VUS	0.0091	.	0.000599	0.003	0.0001	0	0	0.0015	0	0	0	0		
TG	NM_003235:c.C7753T:p.R2585W	1	missense	reported	VUS	0.0091	.	.	.	9.89E-05	0	0	0	0	0.0002	0	0		
TG	NM_003235:c.C2183A:p.T728K	1	missense	unreported	VUS	0.0091	.	.	.	3.30E-05	0	0	0.0003	0	0	0	6.06E-05		
TG	NM_003235:c.T8296A:p.Y2766N	1	missense	reported	VUS	0.0091	.	.	.	3.30E-05	0	0	0.0005	0	0	0	0		

TG	NM_003235:c.G7411T:p.A2471S	1	missense	unreported	VUS	0.0091	.	0.0003994	0.002	4.12E-05	0	0	0.0006	0	0	0	0		
TG	NM_003235:c.C1175T:p.P392L	1	missense	reported	VUS	0.0091	0.0003	.	.	0.0003	0	0	0.0001	0.0002	0.0006	0.0011	0		
TSHR	NM_000369:c.G1349A:p.R450H	2	missense	reported	Pathogenic	0.0182	.	0.0007987	0.004	0.0003	0	0	0.0044	0	4.50E-05	0	0	loss of function	Cassio A, et al.J Clin Res Pediatr Endocrinol. 2013
TSHR	NM_000369:c.G1576A:p.A526T	1	missense	reported	VUS	0.0091	.	0.0003994	0.002	0.0002	0	0	0.0025	0	3.00E-05	0	0		
TSHR	NM_000369:c.T647C:p.I216T	1	missense	reported	VUS	0.0091	.	0.0001997	.	1.66E-05	0	0	0	0	3.01E-05	0	0		
TSHR	NM_000369:c.G823A:p.A275T	1	missense	reported	VUS	0.0091	.	.	.	0.0001	0	0	0.0015	0	1.65E-05	0	6.66E-05		
TSHR	NM_000369:c.G394C:p.G132R	1	missense	reported	Pathogenic	0.0091	.	0.0011981	0.001	0.0004	0.0014	0.0003	0.0006	0	0.0002	0	0.0005	loss of function	Cassio A, et al.J Clin Res Pediatr Endocrinol. 2013
TSHR	NM_000369:c.A2146G:p.S716G	1	missense	unreported	VUS	0.0091	.	0.0007987	0.004	0.0003	0	0	0.0044	0	4.50E-05	0	0		
TSHR	NM_000369:c.	1	misse	rep	VUS	0.0	.	.	.	4.9	0	0	0.0	0	1.5	0	0		

	G326A:p.R109Q		nse	orted		091				4E-05			006		0E-05				
TSHR	NM_000369:c.A1115C:p.N372T	1	missense	unreported	VUS	0.0091	.	0.000599	0.003	0.0003	0	0	0.0038	0	0	0	0		
TSHR	NM_000369:c.A2252G:p.K751R	1	missense	reported	VUS	0.0091	.	.	.	4.12E-05	0	0	0.0006	0	0	0	0		
TSHR	NM_000369:c.C1582A:p.R528S	1	missense	reported	VUS	0.0091	7.70E-05	.	.	4.94E-05	9.62E-05	0	0.0003	0	1.50E-05	0	6.06E-05		
TPO	NM_000547:c.2268dupT:p.C756fs	4	frameshift insertion	reported	pathogenic	0.0364	7.70E-05	0.0001997	.	1.65E-05	9.61E-05	0	0	0	1.50E-05	0	0	loss of function	Lee CC, et, al.Biomed Res Int. 2014
TPO	NM_000547:c.G1327C:p.A443P	3	missense	reported	Likely pathogenic	0.0273	.	0.0013978	0.0069	0.0004	0	0	0.005	0	0	0	0		
TPO	NM_000547:c.G566A:p.R189Q	1	missense	reported	VUS	0.0091	.	0.000599	0.003	0.0007	0	0	0.0092	0	0	0	0		
TPO	NM_000547:c.T2291C:p.L764P	1	missense	unreported	VUS	0.0091	.	.	.	0.0001	0	0	0.0021	0	0	0	0		
TPO	NM_000547:c.	1	misse	rep	VUS	0.0	.	.	.	4.9	0	0	0.0	0	0	0	0		

	G2578A:p.G860R		nse	orted		091				5E-05			007						
TPO	NM_000547:c.T1282A:p.W428R	1	missense	unreported	VUS	0.0091	.	.	.	8.24E-06	0	0	0.0001	0	0	0	0		
TPO	NM_000547:c.G1465A:p.A489T	1	missense	reported	VUS	0.0091	.	.	.	.	.	.	.	.	.	.	.		
TPO	NM_000547:c.C2647T:p.P883S	1	missense	reported	VUS	0.0091	.	.	.	.	.	.	.	.	.	.	.	gain of function	Yoshizawa-Ogasawara A,et al.J Pediatr Endocrinol Metab. 2016
TPO	XM_005264700.1:c.2735dupT:p.Q913fs	1	frameshift insertion	reported	Likely pathogenic	0.0091	.	.	.	.	.	.	.	.	.	.	.		
DUOXA1	NM_001276268:c.C503T:p.T168M	12	missense	reported	Likely pathogenic	0.1091	.	0.0001997	0.001	3.30E-05	0	0	0.0005	0	0	0	0		
DUOXA1	NM_001276268:c.C166T:p.R56W	1	missense	reported	VUS	0.0091	.	.	.	.	.	.	.	.	.	.	.		
DUOXA1	NM_001276268:c.C601T:p.H201Y	1	missense	unreported	VUS	0.0091	.	.	.	.	.	.	.	.	.	.	.		
DUOXA2	NM_207581:c.	3	stopg	rep	Pat	0.0	.	0.000	0.003	0.0	0	0	0.0	0	0	0	0		

	413dupA:p.Y138_A139delinsX		ain	orted	hogenic	273		599		002			028						
DUOXA2	NM_207581:c.T788C:p.L263P	2	missense	unreported	VUS	0.0182	.	.	.	.	.	.	.	.	.	.	.		
DUOXA2	NM_207581:c.C37T:p.Q13X	2	stopgain	unreported	Pathogenic	0.0182	.	0.0003994	0.002	1.70E-05	0	0	0.0002	0	0	0	0		
DUOXA2	NM_207581:c.C488T:p.P163L	1	missense	reported	VUS	0.0091	.	.	.	.	.	.	.	.	.	.	.		
DUOX1	NM_017434:c.T3435A:p.S1145R	2	missense	reported	VUS	0.0182	.	.	.	.	.	.	.	.	.	.	.		
DUOX1	NM_017434:c.C415A:p.R139S	1	missense	reported	Likely benign	0.0091	.	.	.	.	.	.	.	.	.	.	.		
DUOX1	NM_017434:c.A580G:p.R194G	1	missense	reported	Likely benign	0.0091	.	.	.	5.79E-05	0	0	0.0001	0	0	0	0.0004		
DUOX1	NM_017434:c.C3236A:p.T1079N	1	missense	unreported	VUS	0.0091	.	.	.	1.65E-05	0	0	0	0	3.00E-05	0	0		
DUOX1	NM_017434:c.G3920A:p.R1307Q	1	missense	reported	VUS	0.0091	.	.	.	2.47E-05	0	0	0	0	4.50E-05	0	0		

DUOX1	NM_017434:c.T2957C:p.L986P	1	missense	reported	VUS	0.0091	7.70E-05	.	.	4.12E-05	0	0	0	0	7.50E-05	0	0		
DUOX1	NM_017434:c.A1707T:p.R569S	1	missense	reported	Likely benign	0.0091	0.0002	0.0003994	0.001	0.0002	0.0004	0	0.0015	0	7.50E-05	0	0		
DUOX1	NM_017434:c.C1117A:p.P373T	1	missense	reported	VUS	0.0091	.	.	.	.	.	.	.	.	.	.	.		
DUOX1	NM_017434.3:c.3524+1 -c.3524+4del	1	splicing	reported	Pathogenic	0.0091	.	.	.	.	.	.	.	.	.	.	.		
DUOX1	NM_017434:c.G149A:p.R50Q	1	missense	reported	VUS	0.0091	.	.	.	.	.	.	.	.	.	.	.		
SLC26A4	NM_000441:c.C147G:p.S49R	1	missense	reported	VUS	0.0091	.	.	.	.	.	.	.	.	.	.	.		
SLC26A4	NM_000441:c.919-2A>G	1	splicing	reported	pathogenic	0.0091	.	.	.	8.24E-06	0	0	0	0	1.50E-05	0	0		
SLC26A4	NM_000441:c.A1087C:p.I363L	1	missense	unreported	VUS	0.0091	.	.	.	.	.	.	.	.	.	.	.		
SLC26A4	NM_000441:c.A2168G:p.H723R	1	missense	reported	VUS	0.0091	.	.	.	.	.	.	.	.	.	.	.		
GNAS	NM_016592:c.G838A:p.A280	1	missense	report	VUS	0.009	.	.	.	1.69E	0	0	0.000	0	0	0	0		

	T			ed		1				-05			2						
GNAS	NM_016592:c.C205A:p.H69N	1	missense	reported	VUS	0.0091	.	0.0017971	.	0.0046	0.0014	0.0095	0	0.0119	0.0092	0.0195	0.0017		
GNAS	NM_016592:c.G334A:p.E112K	1	missense	reported	VUS	0.0091	.	.	.	.	.	.	.	.	.	.	.		
PAX8	NM_013952:c.C1037T:p.P346L	3	missense	reported	pathogenic	0.0273	.	.	.	.	.	.	.	.	.	.	.		
HHEX	NM_002729:c.A199G:p.T67A	1	missense	unreported	VUS	0.0091	.	0.0003994	0.001	0.0002	0.0002	0	0.0029	0	0	0	6.06E-05		
FOXE1	NM_004473:c.C334T:p.L112F	1	missense	reported	VUS	0.0091	.	.	.	0.0003	0	0	0.0042	0	0	0.0011	0		
FOXE1	NM_004473:c.C23G:p.P8R	1	missense	unreported	VUS	0.0091	.	0.0013978	0.0069	0.0006	0	8.65E-05	0.0074	0	0	0.0011	0.0002		
NKX2-1	NM_001079668:c.G1054A:p.G352S	2	missense	reported	VUS	0.0182	.	0.0001997	0.001	2.55E-05	0	0	0.0002	0	0	0	6.76E-05		
IYD	NM_001164694:c.A706G:p.M236V	1	missense	unreported	VUS	0.0091	.	0.0017971	0.0079	0.0005	0.0009	0	0.0035	0	0.0001	0.0022	0.0003		
THRA	NM_001190918:c.A508G:p.I170V	1	missense	reported	Likely beni	0.0091	7.70E-05	0.0001997	.	4.59E-	0.0001	8.74E-05	0.0001	0	0	0	0.0002		

					gn					05									
VUS,variants uncertain significance																			
