# Supplementary material for: The genetic characteristics of congenital hypothyroidism in China by comprehensive screening of 21 candidate genes
Source: Eur J Endocrinol. 2018 Mar 28;178(6):623–33. doi: 10.1530/EJE-17-1017 (PMC5958289; doi:10.1530/EJE-17-1017)
Supplement: Supporting Table 3 [file eje-178-623-t003.pdf]

Supplemental Table 3. Thyroid function of 22 pedigrees from figure 4.

	TSH (uIU/ml)	FT3 (pg/ml)	FT4 (ng/dl)
1-1	29.07	4.25	2.66
1-1-F	2.19	3.56	0.74
1-1-M	1.67	3.90	0.97
1-2	130.53	3.91	0.57
1-2-F	0.83	3.77	0.69
1-2-M	1.06	3.34	0.72
2-1	91.11	2.20	0.29
2-1	NA	NA	NA
2-1/2-2-F	0.70	3.46	0.96
2-1/2-2-M	4.00	3,23	0.66
3	20.01	3.06	0.97
3-F	1.50	3.47	1.00
3-M	2.09	4.12	1.05
4	100.00	2.19	0.28
4-F	0.85	3.43	0.84
4-M	1.35	3.30	0.89
5	8.88	3.66	1.30
5-F	2.09	4.12	1.05
5-M	3.62	3.71	0.96
6	69.64	2.42	0.50
6-F	0.85	3.43	0.84
6-M	1.35	3.30	0.89
7	>100	1.48	0.20
7-F	2.01	3.60	0.84
7-M	1.40	3.73	0.89
8	>100	2.03	0.38
8-F	1.60	3.31	0.68
8-M	1.06	3.56	0.74
9	150.00	NA	NA
9-F	4.37	4.25	0.84
9-M	2.01	3.94	0.85
10	NA	NA	NA
10-F	1.16	3.71	1.00
10-M	3.20	3.35	0.77
11	16.87	NA	NA
11-F	2.00	3.98	0.88
11-M	5.02	3.27	0.68
12	>100	1.81	<0.4
12-F	1.00	11.46	1.47
12-M	1.16	3.33	1.18
13	NA	NA	NA
13-F	2.76	3.99	0.94
13-M	1.87	3.71	0.83
14	>150	1.44	0.25
14-F	4.38	3.70	0.90
14-M	5.20	3.19	0.80

	TSH (uIU/ml)	FT3 (pg/ml)	FT4 (ng/dl)
15	9.62	3.99	1.12
15-F	0.44	2.98	1.14
15-M	0.76	4.35	1.03
16	NA	NA	NA
16-F	1.91	3.17	1.12
16-M	0.53	6.20	1.19
17	64.78	3.98	0.72
17-F	1.09	3.52	0.89
17-M	2.45	4.19	0.96
18	NA	NA	NA
18-F	1.96	3.43	0.86
18-M	1.37	2.90	NA
19	>100	2.28	<0.4
19-F	1.03	3.53	0.67
19-M	1.65	3.83	0.64
20	150.00	NA	NA
20-F	1.83	3.72	0.78
20-M	1.30	3.15	0.75
21	>150	NA	NA
21-F	3.05	3.44	0.83
21-M	1.58	3.41	0.78
22	32.50	3.66	1.01
22-F	2.28	4.60	0.93
22-M	2.55	3.79	0.91
Ref.values	0.34–5.6	2.0–4.4	0.93–1.7

NA, not available.
