# Supplementary material for: The genetic characteristics of congenital hypothyroidism in China by comprehensive screening of 21 candidate genes
Source: Eur J Endocrinol. 2018 Mar 28;178(6):623–33. doi: 10.1530/EJE-17-1017 (PMC5958289; doi:10.1530/EJE-17-1017)
Supplement: Supporting Table 4 [file eje-178-623-t004.pdf]

Supplemememtal Table 4. Final Summary Matrix Used to Provisionally Classify Gene-CH Associations.

gene	Inheritance Pattern	Points of genetic evidence (0-12 points)	Points of experimental evidence (0-6 points)	Total points (0-18 points)	Replication over time (Y/N)	Valid contradictory evidence	Final classfiation
DUOX2	recessive	12	6	18	Y	N	definitive
DUOXA2	recessive	12	6	18	Y	N	definitive
DUOX1	recessive	2	3	5	N	N	limited
DUOXA1	recessive	2	6	8	Y	N	moderate
TPO	recessive	12	4	16	Y	N	definitive
TG	recessive	12	6	18	Y	N	definitive
TSHR	recessive	12	6	18	Y	N	definitive
SLC5A5	recessive	12	6	18	Y	N	definitive
SLC26A4	recessive	12	6	18	Y	N	definitive
SLC16A2	X-linked	7	5	12	Y	N	definitive
IYD	recessive	12	4	16	Y	N	definitive
THRA	dominant	12	6	18	Y	N	definitive
THRB	dominant	12	4	16	Y	N	definitive
PAX8	N/A	12	6	18	Y	N	definitive
FOXE1	N/A	12	6	18	Y	N	definitive
NKX2-1	N/A	12	6	18	Y	N	definitive
NKX2-5	N/A	12	6	18	Y	N	definitive
HHEX	N/A	0	4	4	N	N	no reported evidence
DIO1	N/A	0	3	3	N	N	no reported evidence
DIO2	N/A	0	3	3	N	N	no reported evidence
GNAS	N/A	7.5	4.5	12	Y	N	strong

Abbreviations: N/A, not applicable.Y, yes. N, no.
